# Microbial transmission and ecology of human and environmental microbial communities in childcare centers

**DOI:** 10.21203/rs.3.rs-10207669/v1

**Published:** 2026-09-10

**Authors:** Di Chen, Ya Wang, Daniel M. Portik, Jacob Nearing, William A. Nickols, Jeremy E. Wilkinson, David C. Christiani, Long H. Nguyen, Eric A. Franzosa, John D. Spengler, Curtis Huttenhower, Kelsey N. Thompson

**Affiliations:** 1 Department of Biostatistics, Harvard T.H. Chan School of Public Health, Boston, MA, 02115, USA; 2 Department of Environmental Health, Harvard T.H. Chan School of Public Health, Boston, MA, 02115, USA; 3 Harvard Chan Microbiome in Public Health Center, Harvard T.H. Chan School of Public Health, Boston, MA, 02115, USA; 4 Infectious Disease and Microbiome Program, Broad Institute of MIT and Harvard, Cambridge, MA, 02142, USA; 5 Pacific Biosciences, Menlo Park, CA, 94025, USA; 6 Pulmonary and Critical Care Division, Department of Medicine, Massachusetts General Hospital and Harvard Medical School, Boston, MA, 02114, USA; 7 Department of Immunology and Infectious Diseases, Harvard T.H. Chan School of Public Health, Harvard University, Boston, MA, 02115, USA; 8 Clinical and Translational Epidemiology Unit, Massachusetts General Hospital and Harvard Medical School, Boston, MA, USA; 9 Division of Gastroenterology, Massachusetts General Hospital and Harvard Medical School, Boston, MA, USA

**Keywords:** Early-life microbiome, Childcare microbiome, Built environment, Microbial transmission, Long-read metagenomics, Mobile genetic elements, Lateral gene transfer, Phage ecology

## Abstract

**Background:**

Early-life microbial exposures profoundly impact lifelong health trajectories by shaping immune maturation and modulating disease risk, e.g. as described by the "hygiene hypothesis." Childcare facilities represent a critical yet understudied source of microbial exposures where preschool-aged children spend 7-10 hours daily during key developmental periods. This study presents the first multi-omic and multi-kingdom investigation of microbial transmission and community ecology across childcare environments, integrating microbiome samples from both high-touch and low-touch surfaces with children's nasal and oral microbiomes, using full-length 16S rRNA gene and internal transcribed spacer (ITS) amplicon sequencing paired with short-read (SR) and long-read (LR) metagenomic sequencing. This combination provides enhanced species-level taxonomic resolution and improved recovery of genomes and genomic elements compared to conventional short-read approaches.

**Results:**

Our findings revealed distinct microbial signatures across environments, with human-associated microorganisms predominating in high-touch areas, while greater taxonomic diversity characterizes low-touch areas. Specifically, on high-touch surfaces, several bacterial and fungal species were shared between host and environmental communities, such as food-associated *Lactococcus lactis* and *Streptococcus thermophilus*, suggesting defined transmission routes via host shedding, environmental exposures, and food consumption. With improved genomic resolution and reconstruction from paired SR and LR metagenomics, we identified novel lateral gene transfer (LGT) events enriched for mobile elements, DNA-interacting domains, and adaptive elements such as antibiotic resistance and virulence factors. These methods also shed light on viral ecology, such as *Caudoviricetes* bacteriophages ubiquitous across host and environmental communities with phylogenetically-differentiated niche- and bacterial host-specific lineages. The positive relationship between the host prediction frequency and its community relative abundance suggested host abundance (availability)-driven phage-bacteria population dynamics.

**Conclusions:**

This work highlights previously understudied components of early-life microbial exposures in childcare environments with enhanced resolution and provides methods for identifying potential pathogen reservoirs, tracking transmission routes, and developing targeted interventions.

## Introduction

Microbial communities in the built environment (BE) encompass the diverse assemblages of bacteria, fungi, viruses, and other microorganisms that inhabit human-made structures. These often low biomass, but dynamic ecosystems are shaped by complex interactions between architectural parameters, environmental factors (e.g., temperature, humidity, ventilation), and human activity patterns [1]. They play a critical role in the development of human health, with microbial exposures having been linked to immune system modulation, allergy development, and susceptibility to infectious diseases through their influence on indoor air quality, surface microbiomes, and pathogen transmission dynamics [1,2]. Among various BEs relevant to early-life microbial exposure, childcare facilities represent a critical, high-traffic ecosystem. Young children spend significant time in these densely occupied spaces during key developmental periods. Their behaviors – such as hand-to-mouth contact, floor crawling, and frequent sharing of objects and surfaces – facilitate microbial exchange. Because the nature of these environments amplifies microbial impact, several studies have examined microbial composition in childcare settings [3,4,5,6,7]. However, the genomics of BE (including childcare) microbiomes remain largely understudied, leaving open questions surrounding how these microbial communities are shaped through forces such as selection, evolution, and lateral gene transfer.

Microbial exposures during early life, among other environmental exposures, significantly impact children's developing immune systems and long-term health trajectories [8]. Early life is a crucial period for microbiome establishment and immune maturation (usually defined as 2-3 years), beyond which rapid gut microbiota change decelerates [9]. Specifically, the host’s immune system develops in dialogue with microbial exposures [8], requiring diverse and appropriate microbial encounters to establish proper immune tolerance and response mechanisms [8]. Disruptions or perturbations to these early-life microbial exposures in turn can inflate the risk of disease development later in life [8]. This relationship aligns with the "hygiene hypothesis," which postulates that early life microbial exposures are essential for proper immune development [10,11], with evidence suggesting that routine microbial exposure in childcare environments may be protective against allergies [12], asthma [13], and type 1 diabetes [14], among other immune-mediated disorders. Therefore, gaining a comprehensive understanding of microbial exposures in childcare environments is essential for developing evidence-based strategies that create healthier facilities – both minimizing infectious disease transmission and supporting the beneficial development of children's microbiomes and immune systems during this critical developmental period.

Limited studies on the childcare microbiome observe that microbial communities in these particular environments are distinct from residential settings (e.g. home), specifically observing a higher overall diversity and abundance of human-associated bacteria [4,15]. This suggests that childcare facilities could serve as a distinct reservoir for microbial exchange and potential transmission of both beneficial and pathogenic organisms. However, the majority of previous studies have focused on either the indoor airborne [5,16] or floor dust communities [17,18] and have relied predominantly on SR amplicon sequencing [3,5,17,18,19,20]. This is largely due to the low biomass nature of environmental microbiome samples, which often yields insufficient genetic materials for metagenomic sequencing. Using SR amplicons, consequently, often restricts taxonomic resolution to the genus level and hinders detailed analysis of microbial transmission patterns. Moreover, due to the inherent limitations of using a bacterial or fungal specific marker gene, current approaches predominantly overlook important nonbacterial and nonfungal components of these microbial communities. Indeed, members such as viruses and mobile genetic elements (MGEs) also play important roles in shaping microbial ecology [21]. To our knowledge, only a few shotgun metagenomic studies [15,16] have been conducted in childcare settings, and these focused exclusively on airborne bacterial and viral profiling. While previous studies have primarily addressed "who is there" in childcare environments, comprehensive understanding of these microbiomes requires investigation of genomic and functional elements to provide insights beyond taxonomic identification. These limitations leave critical gaps in our understanding of microbial transmission patterns and functional elements that shape community structure and dynamics in these important environments.

Here, we comprehensively investigated microbial ecology of childcare microbial communities integrating multi-platform data from SR and PacBio LR sequencing. The study combines host and environmental microbial community data, using full-length bacterial and fungal amplicons (16S and ITS) and paired SR and LR shotgun metagenomics, which in combination provide enhanced taxonomic resolution and the ability to assess functional elements and evolutionary signatures across environments. We specifically investigated microbial transmission patterns, mobile genetic elements and their biochemical and regulatory functionality, lateral gene transfers, and phage-host interactions. This study is thus the first to elucidate previously understudied components of early-life microbial exposures in childcare environments and offers a more comprehensive view of microbial diversity and functional potential within these communities. This work also provides methods for identifying potential pathogen reservoirs, tracking transmission routes, and developing targeted interventions in childcare settings.

## Results

### High-resolution taxonomy of childcare microbiomes by combining multiple sequencing platforms and techniques

We collected a total of 177 environmental and host microbial community samples from two university-affiliated childcare centers (Fig. 1A). Host samples (n = 68) were cross-sectionally collected by swabbing the oral and nasal cavities of 34 children aged 2 to 4 years. Environmental samples (n = 109) were collected from eight classrooms from a variety of BE types of different touch frequencies, including doorknobs, desks, chairs, toys, carpets, infiltration areas, heating, ventilation and air conditioning (HVAC) systems, sinks, drains, outdoor air, and soil (Methods; Additional files 1 and 3). Sample types were further grouped into two categories based on touch frequency: drain, sink, HVAC, outdoor air, and soil were classified as “low-touch” environments, whereas all other surfaces were classified as “high-touch” environments. We generated full-length 16S rRNA gene (FL-16S) and internal transcribed spacer (FL-ITS) sequence data using PacBio amplicon sequencing for bacterial and fungal community profiling. A subset of samples with sufficient biomass – comprising two individual drain samples, a pooled oral sample, and a pooled soil sample (n = 4) – were subjected to both SR (SR-MGX) and LR shotgun metagenomic sequencing (LR-MGX). Per-sample-type counts are summarized in Additional file 1, and full per-sample metadata are available in Additional file 2.

We identified 22,455 bacterial amplicon sequence variants (ASVs) from the FL-16S sequencing (Additional file 3) and 1,097 fungal ASVs from FL-ITS (Additional file 3), yielding 583 bacterial and 414 fungal species assignments. The unusually high number of ASVs appeared to be a product of current published full-length amplicon pipelines being overly permissive for low-frequency sequence variants, given the inherent intragenomic diversity of FL-16S and ITS sequences. To mitigate this, we clustered ASV sequences at 97% identity, yielding 4,381 bacterial (Additional file 3) and 745 fungal (Additional file 3) species-level operational taxonomic units (OTUs) (see Methods).

Even after this additional quality control, the full-length amplicons achieved improved taxonomic resolution when compared to typical short marker gene amplicons, especially in environmental microbial samples. Compared to in silico extracted 16S V4 amplicons (the most commonly used SR target regions), full-length amplicons showed comparable resolution at higher taxonomic levels but increased the ability to assign taxa from the family level and below (Fig. S1). For species-level assignment, full-length 16S amplicons resolved 35.9% of total ASVs, compared to only 6.0% by the V4 amplicons. The improvement was greater in environmental samples (median, 36.6%) than in host samples (median, 17.4%) (Fig. 1D), aligning with previous evaluations that sequencing the entire 16S rRNA gene provides substantial advantages over targeting subregions [22]. This improvement was particularly beneficial in our mixed human/BE for capturing species diversity more accurately and identifying underrepresented taxa.

### Distinct microbial communities identified across human-association gradients

Collection source heavily influenced community diversity and structure, as expected. Alpha diversity of bacterial communities differed significantly among sample types (Kruskal-Wallis p < 0.001) and was highest in soil samples (median Shannon index = 6.99), followed by oral, infiltration, and drain samples (Fig. S2A). Most high-touch environments showed moderate bacterial diversity, with toy samples presenting the lowest within-sample diversity (median Shannon index = 1.90). In contrast, fungal biomass was generally low in indoor settings, with only 61 of 177 samples yielding sufficient DNA for amplification and sequencing. Among these, soil samples showed the highest fungal diversity, followed by carpets - which are a significant reservoir of indoor fungi [23] - while the lowest diversity was observed in sink and drain samples. Since outdoor air is the primary source of indoor fungi [24], the overall limited fungal detection is likely due to mechanical ventilation in both sampling facilities, which filtered out most outdoor fungi from incoming air [24]. However, all of our HVAC samples failed to amplify sufficient DNA for sequencing, not allowing us to test this hypothesis. Bacterial taxonomy showed the greatest dissimilarity between nasal, oral, and environmental communities (Fig. 1B; Bray-Curtis PERMANOVA R^2^ = 0.43; p = 0.01). Notably, incorporating phylogenetic distance (weighted UniFrac) revealed that nasal and oral communities were most similar to high-touch environmental samples (Fig. 1C; Bray-Curtis PERMANOVA R^2^ = 0.25; p = 0.01), which we hypothesized indicated that these communities contained more host-associated bacteria compared to low-touch environments.

Microbial community composition revealed distinct patterns across environments that were driven primarily by the degree of human contact. Host-associated communities were unsurprisingly dominated by well-characterized human commensals: nasal samples contained typical inhabitants including *Staphylococcus sp.* (OTU68: mean abundance, 36.1%), *Dolosigranulum pigrum* (OTU4: 20.1%), and *Corynebacterium sp.* (OTU1168: 9.8%), while oral samples harbored characteristic microbiota such as *Streptococcus spp.* (OTU21 and OTU41: 19.3% and 12.5%, respectively), *Haemophilus sp.* (OTU22: 10.3%), and *Rothia mucilaginosa* (OTU384: 8.0%). High-touch surfaces exhibited the predicted dual signature of human and environmental microbes, harboring skin commensals likely transmitted through direct contact – including *Cutibacterium sp.* (OTU2850: prevalence, 45.2%) and *Micrococcus luteus* (OTU157: prevalence, 19.4%) – alongside prevalent environmental taxa, such as chloroplast sequences from *Cyanobacteria* (OTU1128 and OTU1962: mean abundance, 12.3% and 5.7%, respectively) possibly from indoor plants or outdoor sources, and *Friedmanniella sp.* (OTU889: 3.4%), and *Pseudomonas sp.* (OTU555: 9.6%), both generalists with diverse metabolic capabilities [25].

In contrast, low-touch environments showed minimal human influence and were instead dominated by niche-specific environmental communities. Drain and sink samples, sharing similar biochemical conditions, were co-dominated by methylotrophic *Alphaproteobacteria* including *Methylobacterium-Methylorubrum* species (OTU1805 and OTU174: >40% prevalence) and hydrocarbon-degrading *Sphingomonas paucimobilis* (OTU9) and related *Sphingomonadaceae* (OTU1135: >35% prevalence). Soil samples contained distinct core community members essential for nutrient cycling, including *Solirubrobacterales* 67-14 (OTU244), *Burkholderiales* SC-I-84 (OTU117), *Devosia* (OTU435), and *Hyphomicrobium* (OTU1497). Among samples that had equivalent metagenomes – including two drain, one pooled oral, and one pooled soil sample – about one-third of species detected in amplicon data were also identified by shotgun metagenomics (11-32% shared species) (Fig. S3). This overlap is likely substantially underestimated, however, due to differences in the taxonomic systems available between amplicon and shotgun reference databases. This will disproportionately affect these samples, which are enriched for undercharacterized and often unnamed clades. Nevertheless, this concordance allowed for genome-resolved investigations of these microbes.

### Unique phylogenetic distribution of bacteria and fungi across host and environments indicative of transmission patterns

To explore the taxonomic diversity and relationships between microbial communities in various environments, we reconstructed phylogenetic relationships from full-length 16S and ITS representative ASV sequences (Fig. 2A). This revealed distinct phylogenetic distribution patterns that mirror transmission dynamics. Among 22,455 FL-16S ASVs, 1,229 (5.5%) and 11,658 (51.9%) were identified in nasal and oral samples, respectively, and 1331 (5.9%) and 9,179 (40.9%) ASVs were detected in high-touch and low-touch environments. Host-associated communities presented bacterial membership consistent with previously characterized nasal and oral microbiota [26], dominated by *Firmicutes* (nasal: 46%; oral: 52%), *Proteobacteria* (nasal: 30%; oral: 20%), *Actinobacteria* (nasal: 12%; oral: 14%), and *Bacteroidota* (nasal: 7%; oral: 8%). Low-touch environments exhibited higher diversity, with ASVs largely distinct from host samples. These environments were primarily dominated by *Proteobacteria* (55%) and *Actinobacteria* (16%), alongside the phyla *Planctomycetota* (10%), *Myxococcota* (3%), *Acidobacteriota* (2%), and *Chloroflexi* (2%), all of which are microbes ubiquitously found in natural habitats. In contrast, high-touch environments closely resembled host-associated community structures, with an additional presence of *Cyanobacteria* (3%) likely due to deposition and transportation from indoor plants.

The fungal phylogenetic tree based on FL-ITS ASVs (Fig. 2B) contained fewer tips due to the limited detection of fungal taxa across samples, with the greatest fungal diversity concentrated in environmentally derived communities. Among 1,097 full-length ITS ASVs, 189 fungal ASVs were identified in nasal samples and 28 in oral samples, while 432 and 615 ASVs were detected in high-touch and low-touch environments, respectively. Nasal and oral communities shared similar fungal phyla, predominantly represented by *Ascomycota* (nasal: 61.9%; oral: 46.4%), *Basidiomycota* (nasal: 28.6%; oral: 17.9%), and *Chytridiomycota* (nasal: 4.8%; oral: 3.6%). Both *Ascomycota* and *Basidiomycota* contain known human-associated yeasts such as the genera *Candida* and *Malassezia*, respectively; whereas *Chytridiomycota* are primitive zoosporic fungi commonly found in water and soils, suggesting exposure via inhalation of aerosolized spores and/or direct contact. The distribution in high-touch environments closely resembled nasal fungal communities but with greater ASV diversity within each phylum, suggesting combined contributions from host-associated fungi and those of environmental origin.

In low-touch environments, we identified eight fungal phyla, with *Ascomycota* and *Basidiomycota* remaining the most abundant, followed by *Chytridiomycota* (9%), *Rozellomycota* (4%), and *Mucoromycota* (2%). *Rozellomycota* (also referred to as *Cryptomycota*) are known as the “dark matter” of fungi; despite being ubiquitous and abundant in various aquatic and soil habitats, the strain diversity, let alone the functions of this group remains poorly understood [27]. This is evident in our study, where the majority of *Rozellomycota* ASVs were classified mostly as *Rozellomycota* cls Incertae sedis and a few as *Rozellomycotina* cls Incertae sedis, and their lower-level taxonomy remains unknown. Together, the phylogenetic distribution of bacterial and fungal communities directly reflects the degree of human-environment interactions in the childcare setting, distinguishing high-touch surfaces as human microbiome reservoirs from low-touch surfaces dominated by environmental taxa.

### Transmission of microbes from hosts to surrounding environments

Next, we searched for ASVs that were shared between host and environmental samples, thus indicative of potential transmission events (Fig. 2, Additional file 4). Within the 16S data, 201 bacterial ASVs from oral communities and 177 from nasal communities were shared in high-touch environments (Fig. S4), suggesting possible transmission of human-associated bacteria to indoor surfaces through frequent and repetitive contact. In contrast, low-touch environments only shared 41 and 92 bacterial ASVs with oral and nasal communities, respectively, likely due to limited human contact.

Bacteria shared across host and environmental samples appeared to be either host- or food-associated species, with their highest abundance in host or high-touch samples (Fig. 3A, Additional file 5). Bacterial species most abundant in host samples were largely body-site-specific ASVs, but a few human skin and upper respiratory tract commensals, including *Cutibacterium acnes* (25.0%), *Lautropia mirabilis* (11.1%), *Staphylococcus aureus* (7.1%), and *Staphylococcus epidermidis* (5.6%), had a notable proportion of ASVs universally shared across environmental communities. In contrast, most species with a higher proportion of universally shared ASVs (27% to 70%) were most frequently found in high-touch environments, including *Lactococcus lactis* (66.7%), *Rothia dentocariosa* (33.3%), and *Streptococcus thermophilus* (27.3%). *Sphingomonas echinoides* and *Bradyrhizobium japonicum* were also observed to be highly prevalent and shared across samples, although they may be laboratory contaminants, as previously reported [28]. Species lacking shared ASVs in high-touch environments included *Bifidobacterium adolescentis*, a gut anaerobe occasionally found in probiotic functional foods (Additional file 5), along with various environmental organisms. Finally, bacterial species most abundant in low-touch environments were almost exclusively environmental ASVs.

Two lactic acid bacteria found in high-touch environments - *Lactococcus lactis* and *Streptococcus thermophilus* - are both facultative anaerobes commonly used in food fermentation, such as in dairy products and fermented vegetables. To investigate the potential source of these food-associated organisms, we compared their ASV sequences against reference sequences from dairy products, fermented foods, and non-food-associated environments. The phylogenetic analyses revealed greater similarity to sequences isolated from food sources, including dairy and non-dairy fermented foods, than to non-food-associated ones (*L. lactis*: mean distance to food 0.01 vs. non-food 0.03, Wilcoxon rank sum test p < 0.001; *S. thermophilus*: 0.02 vs. 0.05, p = 0.12) (Fig. 3C). This finding suggests that food consumption, alongside direct contact and host shedding, may be a key route of microbial transmission in the childcare environment.

Fungal ASVs were primarily shared between nasal and environmental communities, with 65 ASVs overlapping in high-touch environments and 41 in low-touch environments, while minimal overlap was observed with oral communities. These findings suggest that fungal transmission is likely driven by nasal inhalation of airborne microorganisms from indoor and outdoor sources, whereas oral transmission, whether through droplets or other means, appears less frequent – possibly reflecting the greater capacity of the nasal cavity to harbor diverse environmental fungi as the primary site of airborne spore deposition [29,30] (Fig. 3B). *Aureobasidium pullulans*, a generalist fungus found in various environments like soil, water, and air, was the only species universally shared across host and environmental communities. Most shared species were observed between nasal communities and environmental samples, with the proportion of shared ASVs ranging from 5% up to 100%, while minimal overlap was observed with the limited oral samples. Many of these fungi are common mold species previously identified in indoor environments, including *Penicillium* (*P. oxalicum*, *P. corylophilum*), *Mucor fragilis*, *Cladosporium* (*C. herbarum*, *C. halotolerans*), *Aspergillus* (*A. fumigatus*, *A. penicillioides*), and *Alternaria* (*A. tenuissima*), likely transmitted via inhalation and fomite interactions [23]. Additionally, several species of *Malassezia* (*M. restricta*, *M. globosa*), common on human skin, were shared between nasal and environmental samples. Other detected fungi included *Paecilomyces variotii* and *Filobasidium magnum*, common environmental contaminants previously found in indoor air and house dust [20]. *Saccharomyces cerevisiae* (associated with baked goods and fruit skins) and *Agaricus campestris* (a mushroom species), were detected across host, high-touch, and sink samples, likely reflecting their link to food consumption. Lastly, *Malassezia pachydermatis*, a zoophilic commensal yeast commonly found on canine skin, was abundant in high-touch environments, suggesting potential transport from household pets.

### LR shotgun metagenomes improve genomic recovery and resolution

We next assessed the LR metagenomes generated for a subset of samples, specifically two drain samples, one pooled oral sample, and one pooled soil sample. Sample pooling was necessary in some cases to obtain sufficient high molecular weight DNA for LR-MGX. In combination with paired SR metagenomes, these are particularly amenable to MGX assembly, in many cases yielding high-quality metagenome-assembled genomes (MAGs). SR and LR sequencing depth was comparable across platforms for all samples except for Drain 7, which required an additional DNA repair step for PacBio HiFi sequencing, resulting in lower read recovery for this sample’s long reads (Table 1). After this, we obtained a mean of 7.6 Gb (59.4M reads) of short-read and 5.2 Gb (0.75M reads) of long-read sequence data per sample, with long reads averaging 7,164 bp in length (range: 5,076–9,313 bp).

We compared multiple assembly strategies for the joint SR/LR data, including SR-only, LR-only, and hybrid approaches. We used two types of hybrid assembly approaches: SR-first assemblers (OPERA-MS [31], Table 2, and hybridSPAdes [32]) and LR-first assembly followed by SR polishing using Polypolish (Additional file 6). As expected, at comparable sequencing depths, SR alone assembles more but shorter contigs (fewer contigs ≥ 10 and 50 kbp; Table 2). SR-first hybrid assemblies generated contig size distributions intermediate between SR and LR assemblies (Fig. 4A, Additional file 6), again expected based on first generating high quality SR contigs before extending with long reads [31,33,34]. Polishing of LR assemblies with short reads, on the other hand, showed minimal improvement over LR alone, as individual contig accuracy as reported by Polypolish consistently exceeded 99%. These also had substantially higher N50, lower L50 values, and the largest individual contigs across all four samples compared to SR and hybrid approaches. Alignment of short reads back to each set of assembled contigs revealed substantially higher alignment rates for both LR and hybrid assemblies, indicating improved assembly quality. These results support prior results for PacBio LR sequencing [35,36], suggesting that it can provide complementary high-quality information alongside FL amplicons even in low-biomass communities.

We continued analysis using 123 medium- and high-quality MAGs from the joint SR/LR assemblies, specifically SR assemblies using MEGAHIT and LR assemblies using hifiasm-meta (Fig. 4B). These included 63 medium- and 60 high-quality MAGs from four samples that clustered into 85 unique genome bins: 62 species-level genome bins (SGBs) (42 known, 20 unknown), 20 genus-level genome bins (GGBs) (13 known, 7 unknown), and 3 known family-level genome bins (FGBs) (Fig. 4C, Additional file 6). Notably, 57 out of 123 MAGs assigned to the same SGBs (spanning 25 SGBs with more than one genome bin) likely represent diversity below the species level, suggesting that populations within these distinct environments harbor intraspecies genomic variation that may reflect niche adaptation or distinct transmission sources.

The taxonomic distribution of MAGs varied by sampled environment. Drain samples were dominated by *Proteobacteria* (n = 29) and *Actinobacteria* (n = 26), while the oral sample yielded mainly *Actinobacteria* (n = 9), followed by *Firmicutes* (n = 4), *Proteobacteria* (n = 4), and *Bacteroidota* (n = 3). From the soil sample, only 4 MAGs were recovered from LR assembly and classified as unknown genera within the family *Conexibacteraceae* (phylum *Actinobacteria*). Among 42 MAGs assigned to kSGBs, we identified 13 unique species that were either undetected or not resolved to the species level using 16S sequencing. This highlights the added resolution of shotgun metagenomics in capturing taxonomic diversity that amplicon-based approaches may miss. Together, these results demonstrate that LR metagenomics substantially improved both assembly contiguity and genomic resolution, recovering high-quality genomes that enable investigation of functional genomic features beyond the scope of amplicon-based methods.

### Mobile genetic elements (MGEs) reveal LGT-enriched molecular functions

MGEs - including plasmids, laterally transferred genes, and other mobilizable elements - are key drivers in bacterial evolution, as they accelerate adaptation and reshape community dynamics, with significant implications for human health [38]. MGEs can transfer antibiotic resistance genes, virulence factors, and metabolic capabilities across microbial communities [38]. To better understand their ecological roles in these communities, we first examined plasmids by classifying contigs as chromosomal, plasmid, or viral based on gene content (viralVerify [39]; Additional file 7).

MGEs were more confidently annotated in LR assemblies, with >55% of contigs receiving definitive annotations (primarily chromosomal), compared to <20% for SR assemblies (Fig. 5A). Comparison of Cluster of Orthologous Groups (COG) [40] functional annotations revealed distinct functional profiles between plasmid and chromosomal contigs (Fig. 5B), and these were consistent between the confidently-annotated subsets of both SR and LR assemblies. As such, subsequent analysis considers both technologies together.

Notably, the resulting total set of plasmid contigs contained a much higher proportion of proteins annotated as hypothetical (SR: 74.0%; LR: 71.0%) compared to chromosomal contigs (SR: 40.0%; LR: 47.6%). This likely reflects a combination of factors: the rapid evolution of plasmid-encoded genes carrying novel adaptive functions, and the underrepresentation of plasmids in reference databases due to their historically lower sequencing and study relative to chromosomal DNA. Despite this high proportion of uncharacterized genes, annotated plasmid-associated functions revealed clear functional specialization. Genes associated with cell motility, the mobilome, and intracellular trafficking/secretion/vesicular transport were significantly enriched in plasmid contigs relative to chromosomal counterparts (Fig. 5B). The observed distinction in functional landscape between plasmid and chromosomal sequences highlight the specialized role of plasmids in mediating genetic exchange and adaptation in these microbial communities.

We next investigated chromosomal lateral gene transfer (LGT) events as another type of genetic mobilization in these communities. We first quantified LGT rates across the four metagenomes using WAAFLE [41] to assess the extent of genetic exchange. Environmental samples (soil and drains) showed similar LGT rates: 0.3-0.5 total events per thousand genes, for which ~25% could determine directionality (Fig. 5C, Additional file 8). In contrast, the oral metagenome exhibited substantially higher LGT rates, in agreement with prior reports of increased HGTs in human-associated communities. This is thought to arise from the close physical proximity of microbes within host-associated niches, particularly structured biofilms like those found in the oral cavity that promote frequent cell-to-cell interactions, with genetic compatibility among closely related host-associated taxa further enhancing successful genetic exchange [42,43].

To characterize LGT mobilized functions, we examined protein domain enrichments in LGT-containing contigs relative to all contigs in each metagenome (Fig. 5D). As expected, LGT-associated genes were strongly enriched for mobile element-related functions. Transposases were among the most consistently enriched functions. Additional mobile element-associated domains included a resolvase N-terminal domain (PF00239), an integrase core domain (PF00665), and a phage integrase family (PF00589). Various DNA-interacting domains were also enriched, including several helix-turn-helix (HTH) variants and transposition-associated ATPases. HTH domains commonly function as DNA-binding transcriptional regulators that control mobile element gene expression, while transposition-associated ATPases provide the energy required for DNA strand separation and recombination during transposition [44]. The enrichment of these regulatory and catalytic functions is consistent with their essential roles in mediating horizontal gene transfer.

Interestingly, we also observed niche-specific molecular functions enriched in individual environments (Fig. 5D). For example, the 'cold-shock' DNA-binding domain (PF00313) helps bacterial cells acclimate to colder growth conditions and was significantly enriched in the soil metagenome. Soil bacteria experience substantial diurnal and seasonal temperature fluctuations [47], and cold-shock proteins enable rapid transcriptional reprogramming in response to temperature stress [48], providing a selective advantage in thermally variable environments. In the oral metagenomes, we found enrichment of lactate/malate dehydrogenase domain (PF02866), suggesting a link to the prevalent fermentative metabolism in oral biofilms. These enzymes are essential for fermentative energy metabolism in the oxygen-limited oral environment and contribute to acidification implicated in dental caries [49], indicating that LGT may disseminate metabolic adaptations among oral bacteria. The drain metagenome enriched several proteins and domains of unknown function. Drain biofilms are expected to experience selective pressures uncommon in well-studied environments, such as chronic low-level biocide exposure from cleaning products, oligotrophic conditions punctuated by nutrient pulses, and colonization of synthetic polymer surfaces [50], thus these unknown functions may reflect genuine genetic innovation. Collectively, these niche-specific functional enrichments suggest that horizontal gene transfer is not stochastic but is instead shaped by local selective pressures, with each environment leaving a distinct ecological imprint on its mobile gene pool.

While antibiotic resistance and virulence factors were not among the most common enriched functions, we did observe a number of specific domains involved in LGT events, including small multidrug resistance proteins (PF00893) and BacA (PF02673), along with multiple secretion system domains that are specialized for the transport of virulence factors in oral and drain metagenomes (Additional file 9). An example oral LGT event (Fig. 5E, top) represents an acquisition of a small multidrug resistance gene flanked by a phage integrase and two other elements of unknown function by a *Streptococcus* species from *Gemella haemolysans*, suggesting a phage-mediated transfer. In a second example from a drain metagenome, the helix-turn-helix domain of a resolvase characterized from *Agrobacterium sp.* ATCC 31749 was detected in an *Agrobacterium tumefaciens* genome, where it integrated between TrbL/VirB6 and VirB8 structural proteins of the Type IV secretion systems that mediate bacterial conjugation and regulatory elements (TraM repressor and LuxR family) [51,52]. This strategic positioning of a DNA-binding domain from site-specific recombinases likely facilitates resolution of DNA intermediates during conjugation, enhancing plasmid transfer and integration efficiency that may confer a selective advantage to recipient organisms in the specific environment.

### Habitat-structured viral diversity and phage-host relationships across environments

Finally, we used geNomad [53] to classify viral contigs from SR and LR assemblies, allowing us to investigate phage-bacterial relationships as another mechanism of genetic mobilization. While the majority (>75%) of viral contigs remained taxonomically unclassified, the identifiable viruses were predominantly bacteriophages spanning four realms: *Duplodnaviria*, *Monodnaviria*, *Varidnaviria*, and *Riboviria* (Fig. 6A, Additional file 10). Most identifiable viruses belonged to class *Caudoviricetes* without further family classification. Comparison of viral profiles between environments revealed distinct compositional differences, predominantly corresponding to enrichments for different typical phage hosts: the oral cavity showed relative enrichment of *Kyanoviridae* (previously part of the morphology-based family *Myoviridae*) (0.19%) and the non-phage eukaryotic viral family *Mimiviridae* (0.15%), with unique enrichment of *Herelleviridae* (phages infecting bacteria in the phylum *Firmicutes* [54]) and *Retroviridae* (family of retroviruses including human endogenous viruses [54]) (both ~0.06%). In contrast, drain environments were enriched with phages from the viral families *Inoviridae* (0.21%) and *Autographiviridae* (an extensively studied family of virulent phages [55]) (0.17%), along with unique enrichment of *Drexlerviridae* (formerly part of the *Siphoviridae*) and *Phycodnaviridae* (algal viruses in aquatic environments). In all of these cases, viral communities were phage-dominated and corresponded to the most common bacterial hosts in each habitat. Human-associated eukaryotic viruses appeared only at low abundance and only in the oral metagenome, consistent with previous observations that most human-associated viromes are predominantly bacteriophage in environments such as the gut [56].

Since the majority of identifiable viruses in our dataset belonged to *Caudoviricetes* - a class of tailed double-stranded DNA phages, one of the best-studied from the human gut [57] - we focused on these phages to analyze virus-host interaction dynamics. We constructed a phylogenetic tree of all sequences belonging to this class and predicted their putative bacterial hosts at the genus level using iPHoP [58] (Fig. 6B, Additional file 10). The tree showed phylogenetically-differentiated lineages across different source environments, suggesting habitat-dependent diversity. *Caudoviricetes* as a class is known to have a broad host range across different environments [59,60]. Based on the host prediction results - which were generally only confident to the phylum level - phages identified in drain environments predominantly targeted bacteria in *Actinobacteria* and *Proteobacteria* (mainly *Alphaproteobacteria*), whereas those in oral environments preferentially targeted *Firmicutes*, *Bacteroidetes*, *Saccharibacteria*, and *Proteobacteria* (primarily *Gammaproteobacteria*). As expected, we observed a strong positive correlation between the frequency of predicted hosts and their relative abundances (ρ = 0.60, p < 0.001) (Fig. 6C, Fig. S5, Additional file 11). These environment-specific host-phage associations, mirroring the relative abundances of the underlying bacterial communities, suggest that *Caudoviricetes* (and other common) phage diversity and host tropism are fundamentally shaped by the ecological context.

## Discussion

This study reveals that childcare microbial communities are organized along a gradient of human contact, with distinct compositional, functional, and ecological signatures across host and environmental niches. We integrated full-length amplicon sequencing with paired short-read and long-read metagenomics, and we applied these across a spectrum of children's oral and nasal microbiomes and diverse environmental surfaces. This identified specific host-to-environment microbial transmission patterns, characterized the functional repertoire of mobile genetic elements (including lateral gene transfer events), and uncovered environment-specific phage ecology (notably strong joint persistence of phage with their hosts). Together, these findings provide the first comprehensive, multi-omic and multi-kingdom view of microbial community structure and ecological dynamics in childcare settings.

These results substantially extend the existing understanding of childcare microbiomes, which have generally focused either on host-associated aspects or airborne or floor dust environmental communities, both generally profiled with short-read amplicon sequencing [5,17,18]. By simultaneously characterizing microbial communities across diverse surface types and children's body sites, we demonstrate that human occupancy structures indoor microbial communities along a contact-frequency gradient. High-touch surfaces harbored a mixture of environmental and host-associated bacteria – including nasal, oral, and skin commensals – while low-touch and outdoor environments showed greater taxonomic diversity dominated by environmental taxa, a pattern confirmed by identifying a substantial proportion of high-touch surface communities to be host-associated commensals. These distinct community structures align with two recent childcare studies [4,15] that reported higher abundance of human-associated bacteria on frequently contacted surfaces.

Similar colonization patterns have been observed in other BEs, where studies of homes [61], elementary schools [62], and urban transit systems [63] have consistently shown that frequently touched surfaces harbor microbes characteristic of human-associated communities, whereas low-touch and outdoor environments maintain their own native community structure [64,65]. These parallel findings across different BEs suggest common principles governing microbial community assembly in human-occupied spaces. Fungal detection was comparatively limited in our study, likely reflecting low fungal biomass in indoor and host samples combined with known ITS amplification biases in certain *Ascomycota* lineages [66,67] and incomplete reference databases. We still identified common indoor molds including *Aspergillus*, *Penicillium*, and *Cladosporium* across environments, consistent with observations in residential homes and particularly dependent on cleaning and moisture exposure patterns [20,68]. Together, these observations raise the question of whether the human-contact gradient reflects active microbial deposition and differential survival on surfaces, or environmental filtering, and what hypotheses might be tested regarding the role of specific surface materials, cleaning regimens, or occupancy patterns in modulating these community structures.

While environmentally-mediated microbial transmission in childcare settings has traditionally been studied from an infection control perspective, understanding nonpathogenic microbial transmission is also important for informing preventive practices that support a healthy environment for children. The only prior study to sample both childcare environments and occupants identified co-occurrence clusters between children's palms and frequently handled objects but did not resolve which species are likely transmitted [15]. By tracking exact 16S sequence variants shared between host and environmental samples, we identified specific transmission signatures: universally shared bacteria were predominantly host- or food-associated, with skin and upper respiratory commensals such as *Cutibacterium acnes*, *Staphylococcus aureus*, and *Staphylococcus epidermidis* showing notable proportions of ASVs shared across environmental communities. Food-associated lactic acid bacteria, including *Lactococcus lactis* and *Streptococcus thermophilus*, were phylogenetically closer to dairy-derived than non-food reference sequences, suggesting that food handling and consumption represent a more significant microbial transmission route in childcare settings than previously recognized [69,70]. These patterns align with growing evidence that shared physical environments are major conduits for microbial transmission, interpersonal routes for which have now been deeply studied [71]. The distinction between universally shared versus environment-specific taxa suggests that certain microbes are more transmissible or persistent on surfaces than others, information that could guide targeted hygiene strategies that reduce pathogen exposure without disrupting beneficial microbial encounters [72,73].

Long-read metagenomic sequencing allowed us to move beyond taxonomic profiling and to address open questions about the ecological forces shaping these microbial communities at the level of mobilized functional elements and evolutionary dynamics. A question of interest in BE microbiology is whether microbial communities on different surfaces simply reflect passive deposition, or whether local selective pressures actively shape community function and evolution. Our analysis of mobile genetic elements provides evidence for the latter: LGT events were not only enriched for canonical mobility functions such as transposases [41] but also carried niche-specific adaptive functions – cold-shock response domains in soil, fermentative metabolism genes linked to oral biofilm acidification, and heavy metal resistance in drains. This pattern of environment-specific functional enrichment suggests that horizontal gene transfer in these communities is shaped by local selective pressures, enabling rapid microbial adaptation to distinct microenvironments within the same building. Plasmid-borne genes were predominantly uncharacterized (~71-74% hypothetical, compared to ~40-48% on chromosomes), consistent with plasmids serving as reservoirs for rapidly evolving, potentially novel adaptive functions that have yet to be catalogued. Although antibiotic resistance and virulence-associated domains were not the most commonly transferred functions, their presence in LGT events – including a phage-mediated acquisition of a multidrug resistance gene by *Streptococcus sp.* from *Gemella haemolysans* in the oral cavity – raises important questions about whether childcare environments may facilitate the horizontal spread of clinically relevant genes among commensal bacteria in close contact with young children.

Several limitations should be noted. Shared ASVs between host and environmental samples are indicative of, but do not confirm, active transmission events – future studies with strain-level resolution and longitudinal sampling will be needed to distinguish ongoing transmission from transient environmental deposition. Additionally, our shotgun metagenomic analysis was restricted to four samples due to the low biomass characteristic of environmental microbiomes, limiting the generalizability of MGE, LGT, and phage findings across the full range of sampled environments. Finally, limited fungal detection across sample types constrained our ability to fully characterize eukaryotic transkingdom interactions in these communities; improved full-length ITS amplification protocols, combined with deeper metagenomic sequencing, will be essential for understanding how fungal, bacterial, and viral communities interact and co-shape the childcare microbiome.

In conjunction with other published investigations, this work advances our understanding of the "childcare microbiome" through novel integration of short- and long-read metagenomics with high-resolution amplicon sequencing and incorporation of host microbial features alongside environmental sampling. Our study bridges environmental and human-associated microbial ecology, revealing important insights into how these microbial communities are formed, maintained, and transferred within childcare settings. Our cross-sectional study focused on university-affiliated childcare centers with stringent sustainability policies and demographically homogeneous populations, providing valuable baseline information about microbial dynamics in these crucial early-life environments.

Future longitudinal studies should expand to diverse childcare settings and demographic groups, incorporating comprehensive sampling of centers, staff, and home environments to further illuminate the complex interactions between human and environmental microbiomes. Such investigations will help identify viable resident microbes and their transmission mechanisms, ultimately informing evidence-based strategies for creating healthier childcare environments given their significant role in early-life microbial exposure.

## Methods

### Sample collection

Two university-affiliated childcare centers participated in this study, with sampling conducted on July 22, 2022. We collected paired nasal and oral microbiome samples from 34 children aged two to four years old across eight classrooms (n = 68). Children were excluded from participation if they had received oral or intravenous antibiotics or chemotherapy within 9 months, reported acute infectious diseases, used topical antibiotics or antifungal applications in the nose or mouth within 1 week, had open wounds in the nose or mouth, experienced infections within 1 week, or underwent surgery involving the nasal or oral cavity within 9 months (excluding routine dental procedures) at the time of sampling. Demographic and health information was collected through parent questionnaires, including children's age, sex, race/ethnicity, early-life factors (breastfeeding, mode of delivery), household characteristics (presence of pets), medical history (antibiotic use prior to age one, medications, respiratory and oral illnesses, hospitalizations), and lifestyle factors (diet, probiotic use, oral hygiene practices). The study protocol and all research activities involving human subjects were approved by the Institutional Review Board at Harvard T.H. Chan School of Public Health (IRB# 22-0579).

Environmental samples (n = 109) were collected from various indoor and outdoor sources across both childcare centers. Indoor samples included doorknobs, desks, chairs, carpet, sinks, drains, infiltration sites (window sills), and HVAC ducts. For infiltration and HVAC duct sampling, 3-10 petri dishes (depending on available space) were placed to collect settling dust and were subsequently pooled. Outdoor samples were collected from two locations per center and included air and soil samples. Outdoor air was sampled using cassettes with 0.45-μm pore-sized MCE filters mounted on tripods 1.5 m above ground, positioned away from pedestrian traffic. Air samplers operated with a vacuum pump at 24 L/min for 24 hours. Soil samples of 0.2 grams of soil were collected from two playground locations per center. All remaining surfaces were sampled using pre-moistened FLOQ swabs (Copan) in autoclaved 0.85% saline, with each surface swabbed thoroughly for one minute. All environmental sampling was conducted on the same day as the collection of children's microbiome samples. All samples were stored on ice for no more than 1 hour before transfer to a central location, where they were stored on dry ice until permanent storage at −80°C.

### Sample extraction and sequencing

DNA was extracted following standardized protocols as described by Comeau et al. [74] and updated by Comeau and Kwawukume (https://www.protocols.io/workspaces/integrated-microbiome-resource-imr/publications) using the AllPrep PowerFecal DNA/RNA Kit (Qiagen). All samples underwent full-length 16S rRNA gene and ITS amplification with PacBio barcode-incorporating primers. The complete 16S rRNA gene was amplified using universal bacterial primers 27F (AGRGTTYGATYMTGGCTCAG) and 1492R (RGYTACCTTGTTACGACTT), while the complete ITS region was amplified using fungi-specific primers ITS1FKYO2 (TAGAGGAAGTAAAAGTCGTAA) and ITS4KYO1 (TCCTCCGCTTWTTGWTWTGC). HiFi sequencing of amplified libraries was performed on the PacBio Sequel IIe system.

A subset of samples (n = 4), comprising two individual drain samples, one pooled oral sample (n = 8 individuals), and one pooled soil sample (n = 4 locations), underwent both Illumina SR and PacBio LR shotgun metagenomic sequencing. SR libraries were sequenced on an Illumina NovaSeq 6000 platform with 2 × 150 bp paired-end configuration, and HiFi sequencing of LR libraries was performed on the PacBio Revio system. Negative controls including a blank swab and a blank filter (at the time of sampling), saline (at the time of sampling), lab air, and reagent control during extraction, were included for contamination identification. Sequencing data from controls that yielded sufficient reads are deposited in the NCBI SRA under BioProject PRJNA1476686; controls that failed to yield detectable DNA are not included.

### Amplicon data processing

Raw full-length 16S data was processed using the HiFi-16S-workflow pipeline (v0.7) (https://github.com/PacificBiosciences/HiFi-16S-workflow.git), which generates high-quality ASVs from PacBio HiFi full-length 16S data and assigns taxonomy using QIIME 2 [75] and DADA2 [76]. Full-length ITS data was processed similarly using the DADA2 ITS pipeline workflow with parameters optimized for full-length amplicons [77]. After quality control, samples with low depth (<200 full-length reads) were excluded, resulting in 109 16S samples (host: n = 61, environmental: n = 48) and 61 ITS samples (host: n = 14, environmental: n = 47) for downstream analysis. ASV feature tables were constructed for both datasets, with taxonomy assigned using the SILVA v138 database [78] for bacteria and the UNITE database (25.07.2023 release) [79] for fungi. ASV frequencies were then sum-normalized to relative abundances (Additional file 3).

After quality control and excluding samples that either failed sequencing (3 FL-16S and 116 FL-ITS) due to insufficient biomass or had low sequencing depth (<200 full-length reads), 109 FL-16S samples (61 host and 48 environmental) and 61 FL-ITS samples (14 host and 47 environmental) were analyzed. Initial processing identified 22,455 bacterial ASVs and 1,097 fungal ASVs, corresponding to 583 and 414 unique bacterial and fungal species, respectively. We observed unusually high ASV diversity across samples; specifically, 83% of 16S ASVs were detected in only a single sample, with relative abundances ranging from <1% to 30%. These singleton ASVs collectively can account for up to 85% of per-sample composition, with similar patterns observed in ITS data. This overestimation of sample diversity was likely due to DADA2 defaults for PacBio amplicons were likely permissive for low-frequency sequences and/or the intragenomic heterogeneity of the multi-copy 16S rRNA gene overestimating sample diversity. To allow more robust between-sample community comparisons while maintaining species-level resolution, we subsequently clustered ASVs into 4,381 bacterial and 745 fungal OTUs at 97% similarity (Additional file 3).

### Amplicon data analyses

Alpha diversity was calculated using the Shannon diversity index implemented in the R package 'vegan' (v2.7.1) [80]. Ordinations were calculated using principal coordinate analysis (PCoA) based on both Bray-Curtis dissimilarity of OTU tables and weighted UniFrac distances of ASVs. Environmental samples were categorized by touch frequency into low-touch (drain, sink, soil) and high-touch (remaining surfaces) environments. For phylogenetic analysis, we performed multiple sequence alignment (MSA) on 16S and ITS ASV sequences separately using MAFFT (v7.515) with default parameters, followed by tree construction using FastTree (v2.1.11) [81]. The resulting phylogenetic trees were visualized using GraPhlAn v1.1.4 [82].

To compare bacterial composition across samples, we plotted the relative abundance of the most abundant and prevalent species by sample type using the R package ComplexHeatmap (v2.24.1) [83]. The resulting subset of species shown in Fig. 1E is the union of the top 5 most abundant and prevalent species identified in each sample type. To estimate ASV contribution for each species, we quantified the presence of ASVs for each species across environmental, oral, and nasal samples, calculating their proportional distribution across these sources. Species shown in Fig. 3A and 3B represent the union of the top 5 most abundant species (per sample type) and most prevalent species (across environmental, oral, or nasal samples).

For the comparison of taxonomic resolution between full-length 16S and the V4 subregion, we generated V4 amplicons by directly trimming the targeted region from the full-length ASV sequences using forward primer 515F (5′-GTGCCAGCMGCCGCGGTAA-3′) and the reverse primer 806R (5′-GGACTACHVGGGTWTCTAAT-3′) using mothur (v1.48.0) [84] pcr.seqs function. The trimmed V4 amplicons were called ASVs and assigned taxonomy using the same DADA2 methodology as above.

### SR metagenomic assembly and MAG construction

We assembled SR metagenomes from the subset samples following the bioBakery workflow (v3.1.0) protocols [85] with a pipeline previously established for SGB construction [86]. Briefly, raw sequencing reads were first processed through KneadData v0.7.7 [87] for quality control, removing adapters, low-quality sequences, and host contamination using the human reference genome hg37 prior to assembly. Following quality control, we assembled reads into contigs (minimum length 2,500 bp) using MEGAHIT 1.2.9 [88]. SR contigs were subsequently grouped into metagenome-assembled genomes (MAGs) using MetaBAT v2.15, based on their GC content and read depth patterns [89]. We then assessed MAG quality using CheckM2 1.0.0 [37], categorizing them as high-quality (>90% completeness, <5% contamination), medium-quality (>50% completeness, <10% contamination), or low-quality (<50% completeness, >10% contamination). After excluding low-quality MAGs, we assigned the remaining high- and medium-quality MAGs into taxonomic groups using PhyloPhlAn 3.0 [90] with the “SGB.Jun23” database. Following previously established thresholds [86], MAGs were assigned to species-level genome bins (SGBs), genus genome bins (GGBs), or family genome bins (FGBs) based on Mash distances of <5%, <15%, or <30%, respectively [86].

### LR metagenomic assembly and MAG construction

LR metagenomes were assembled using hifiasm-meta (v0.6.0) [91] with default settings. MAGs were obtained using HiFi-MAG-Pipeline v2.1.0 (https://github.com/PacificBiosciences/pb-metagenomics-tools/tree/master/HiFi-MAG-Pipeline), which employs a completeness-aware strategy. The pipeline first identifies and bins long contigs >500kb individually, assessing their completeness using CheckM2 [37]. Those with >93% completeness are retained as final MAGs, while <93% complete long contigs are pooled with shorter contigs for further binning using MetaBat2 and SemiBin2 (with LR settings). The bin set is then merged and dereplicated using DAS_Tool, and the final combined set of bins and long complete contigs is evaluated using CheckM2, with quality categories and taxonomy assigned using the same criteria and method as SR MAGs. Additionally, bins containing more than 50 contigs are excluded from further analysis.

### Hybrid metagenomic assembly

We performed hybrid assembly using three complementary approaches: 1) OPERA-MS and 2) hybridSPAdes, which utilize a SR-first strategy by scaffolding SR contigs with long reads, and 3) Polypolish, a SR polishing tool adapted for the LR-first strategy to correct potential small-scale errors in LR assemblies using Illumina reads. Specifically, we used hybridSPAdes [32] 4.0.0 with parameters --meta --pacbio and OPERA-MS [31] v0.9.0 with default settings, and we used Polypolish [92] v0.6.0 in --careful mode to polish the LR assembly with short reads. Assembly quality was evaluated using Quast v5.2.0 [93] across all three assembly types (SR, LR, and hybrid), comparing metrics including total assembly size, contig numbers, largest contig length, N50, and L50. Short reads were mapped to the assemblies using bowtie2 v2.2.3 [94] with --very-sensitive-local mode to assess assembly accuracy.

### Profiling and analysis of novel LGT events

We identified and analyzed novel LGT events from the LR metagenomes using WAAFLE (v1.0) [41]. LGT rates were calculated by normalizing the number of LGT events to the number of background genes in each sample, and events were characterized by their directedness (whether or not the transferring clades could be assigned as donor and recipient). For functional enrichment analysis, we first mapped UniRef90 gene assignments to Pfam domains using HUMAnN 2's utility mapping databases (v0.11.1) [95]. Within each sample, we then quantified the frequency of a given functional domain in both LGT-associated and background genes. The significance of functional enrichment was determined using Fisher's exact test, with the resulting two-tailed p-values corrected within-sample for multiple hypothesis testing using the Benjamini-Hochberg FDR method.

### Identification of metagenome-derived viruses and phage host prediction

Contigs from SR and LR assemblies were classified as plasmid, viral, or chromosomal sequences based on gene content using viralVerify v1.1, a module of metaviralSPAdes [39]. The identified genes from these contigs were additionally annotated with COG functional categories [96] using Prokka 1.14.6 [45].

Viral contigs were annotated using geNomad (v1.9.0) [53] with default parameters to classify viral contigs from SR and LR assemblies. Bacterial host of phages were predicted using iPHoP (v1.3.3) [58] with default parameters. To quantify the relationship between phage host prediction and host abundance, taxonomic profiles were obtained from the SR metagenomes using MetaPhlAn (v4.0.6) with the vOct22_fixed database [97].

A maximum-likelihood phylogenetic tree comprising *Caudoviricetes* contigs longer than 10kb was constructed using MAFFT (v7.515) and FastTree (v2.1.11) [81], as previously described in Amplicon data analysis section. The resulting phylogenetic trees were visualized using GraPhlAn v1.1.4 [82].

## Supplementary Material

1

## Figures and Tables

**Figure 1:**
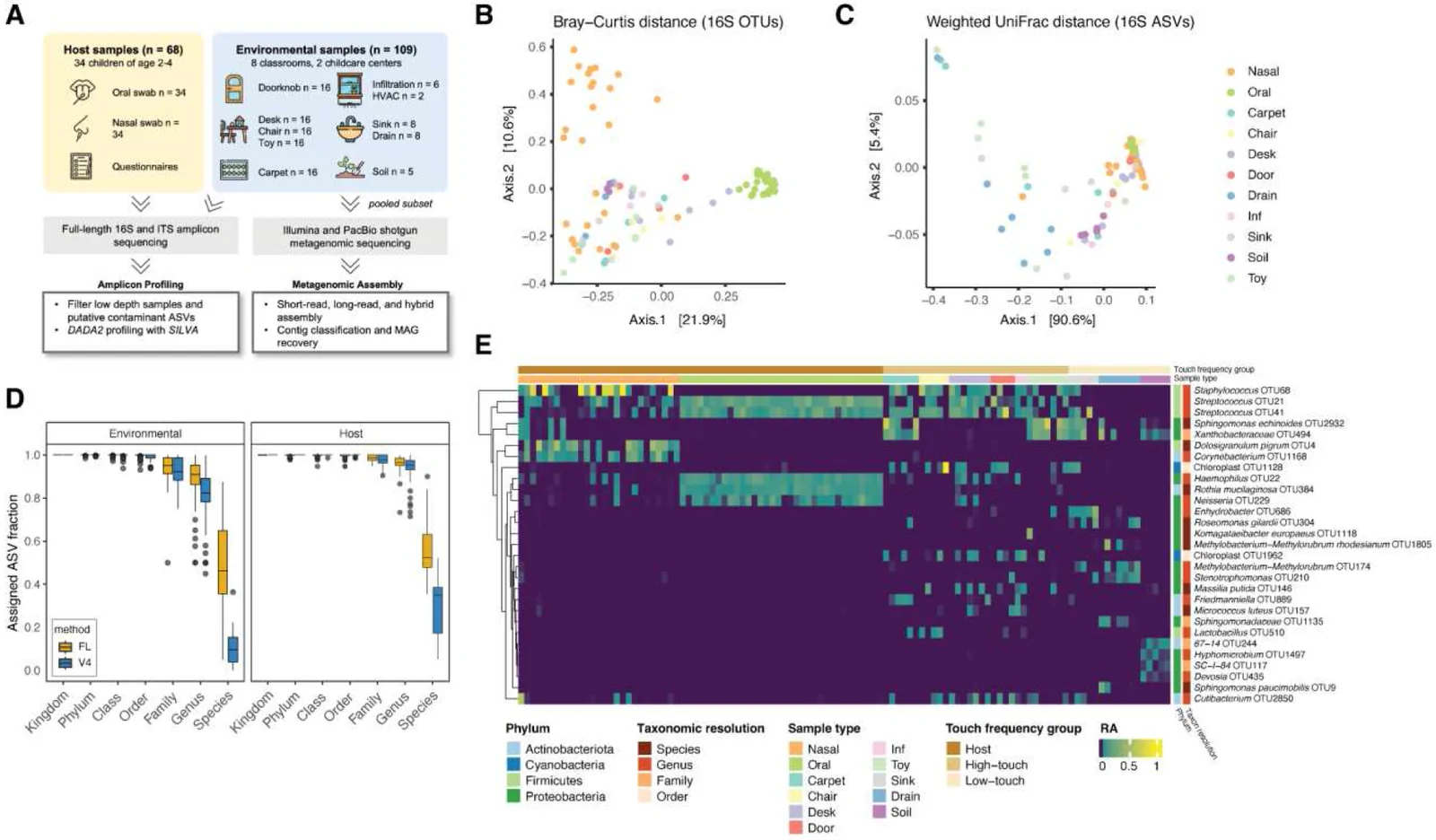
Bacterial compositions across host body sites, high-touch, and low-touch environments. **A)** Study overview. Samples were collected from children's oral and nasal cavities and various environmental surfaces at two different childcare facilities. We employed full-length (FL) amplicon sequencing for bacterial and fungal characterization, along with short read (SR) and long read (LR) shotgun metagenomics to analyze functional elements. Principal coordinate analysis (PCoA) showing sample clustering based on **B)** Bray-Curtis dissimilarities and **C)** weighted UniFrac phylogenetic distances in microbial community composition across sample types. **D)** Species-level taxonomic resolution, measured as the fraction of ASVs assigned at species level, comparing FL and in silico 16S V4 amplicons. **E)** Taxonomic profiles of the most abundant bacteria (29 species representing the union of the 5 most abundant OTUs per sample type). Columns (OTUs) are hierarchically clustered using Euclidean distances. Column annotations indicate key metadata features including sample type and touch frequency. Row annotations denote the phylum-level assignment and the lowest taxonomic assignment level for each OTU.

**Figure 2:**
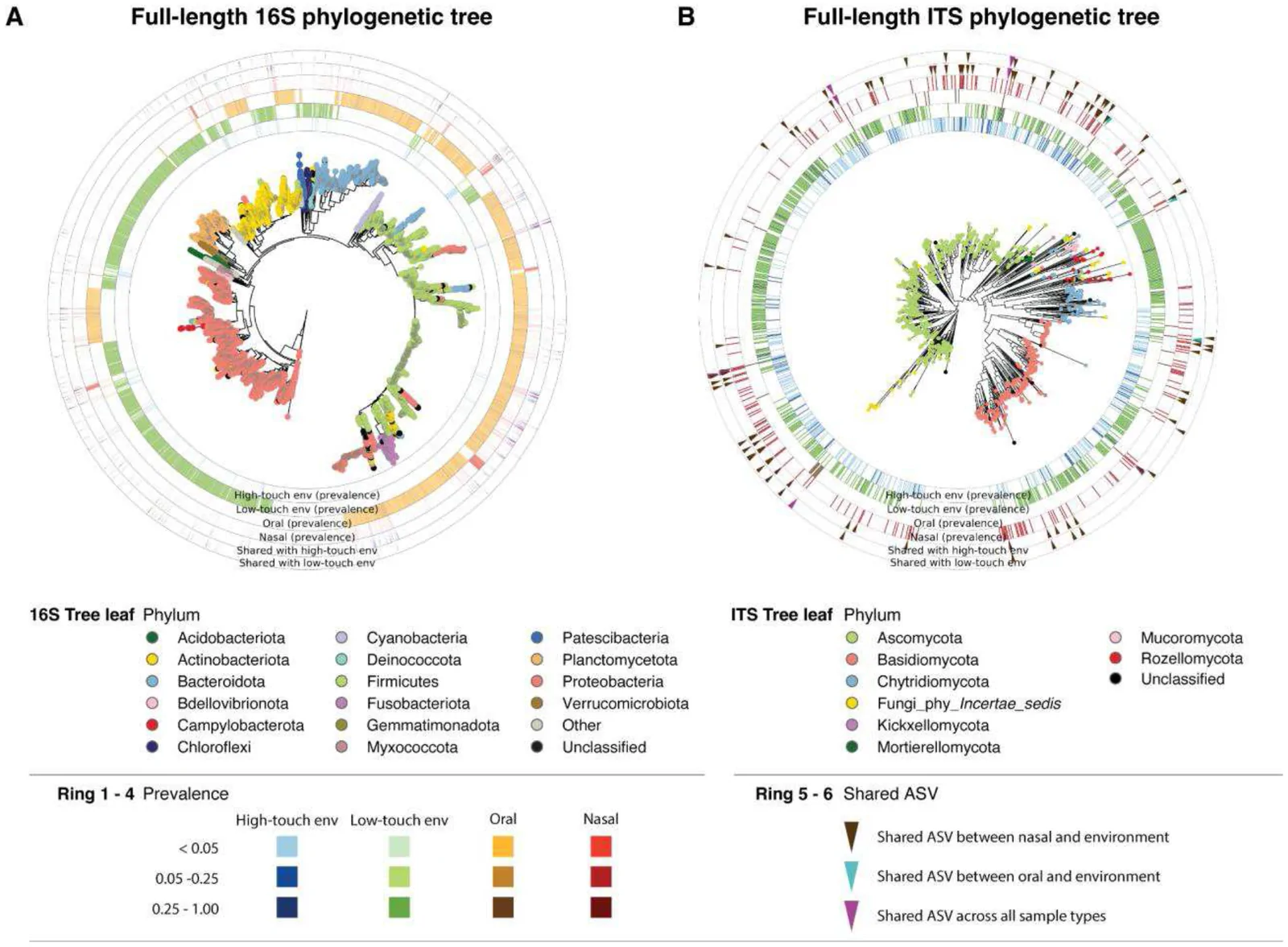
Phylogenetic trees reconstructed from FL-16S and FL-ITS ASVs. Phylogenetic relationships of **A)** 22,455 full-length 16S ASV sequences representing 4,381 bacterial species and **B)** 1,097 full-length ITS ASV sequences representing 735 fungal species. Tree leaves are colored according to phylum-level taxonomic assignment, with bacterial phyla containing fewer than 40 tips grouped as "Other." The inner four rings (rings 1-4) indicate the prevalence of each ASV in respective habitats: high-touch environment, low-touch environment, oral cavity, and nasal cavity. Triangles in the outer two rings (rings 5-6) highlight ASVs shared between host sampling sites (oral/nasal) and environmental surfaces (high-touch or low-touch).

**Figure 3:**
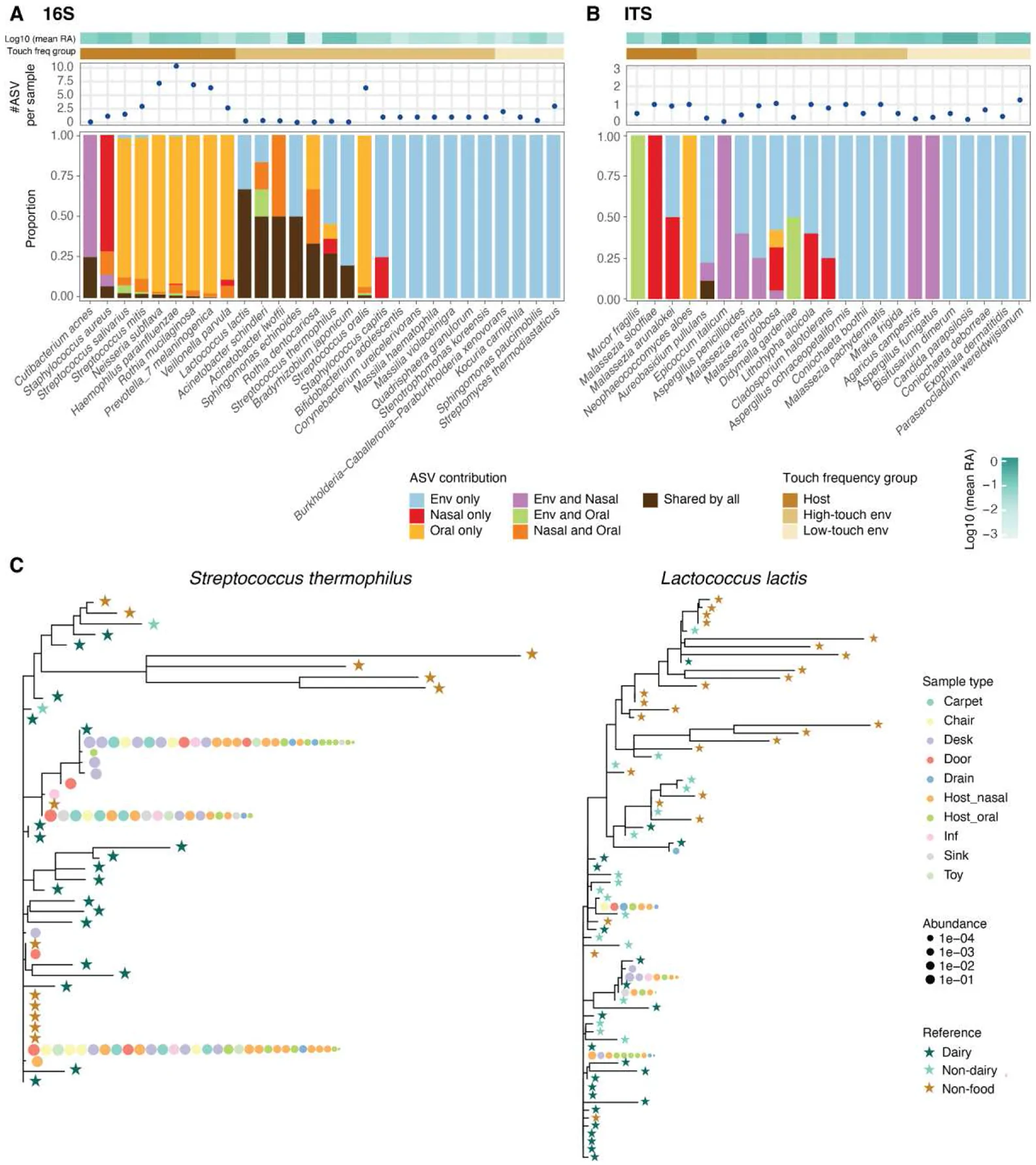
Microbial sharing patterns between hosts and environments reveal potential transmission routes. Stacked bar plot of the relative contribution of ASVs across **A)** 28 bacterial and **B)** 23 fungal species. These species represent the union of the 5 most prevalent species per touch frequency group and the 2 most abundant species per sample type. For each species, bars represent the relative proportion of ASVs detected exclusively in host (“Oral only” or “Nasal only”) or environmental (“Env only”) samples, or shared between any two niches ("Env and Nasal", "Env and Oral", or "Oral and Nasal"), or detected across all sample types (“Shared by all”). This distribution pattern provides insights into potential microbial exchange between children and the childcare environment. **C)** Phylogenetic analysis of *S. thermophilus* (left) and *L. lactis* (right), two species with substantial ASV sharing between hosts and environments. Phylogenetic trees were constructed using representative ASV sequences from this study and reference FL-16S sequences previously isolated from food and non-food sources (retrieved from the NCBI nucleotide database). Phylogenies showed that shared sequences identified in our dataset exhibit closer evolutionary relationships with food-associated reference sequences than with non-food-associated lineages, suggesting dietary sources for the deposited microbes.

**Figure 4:**
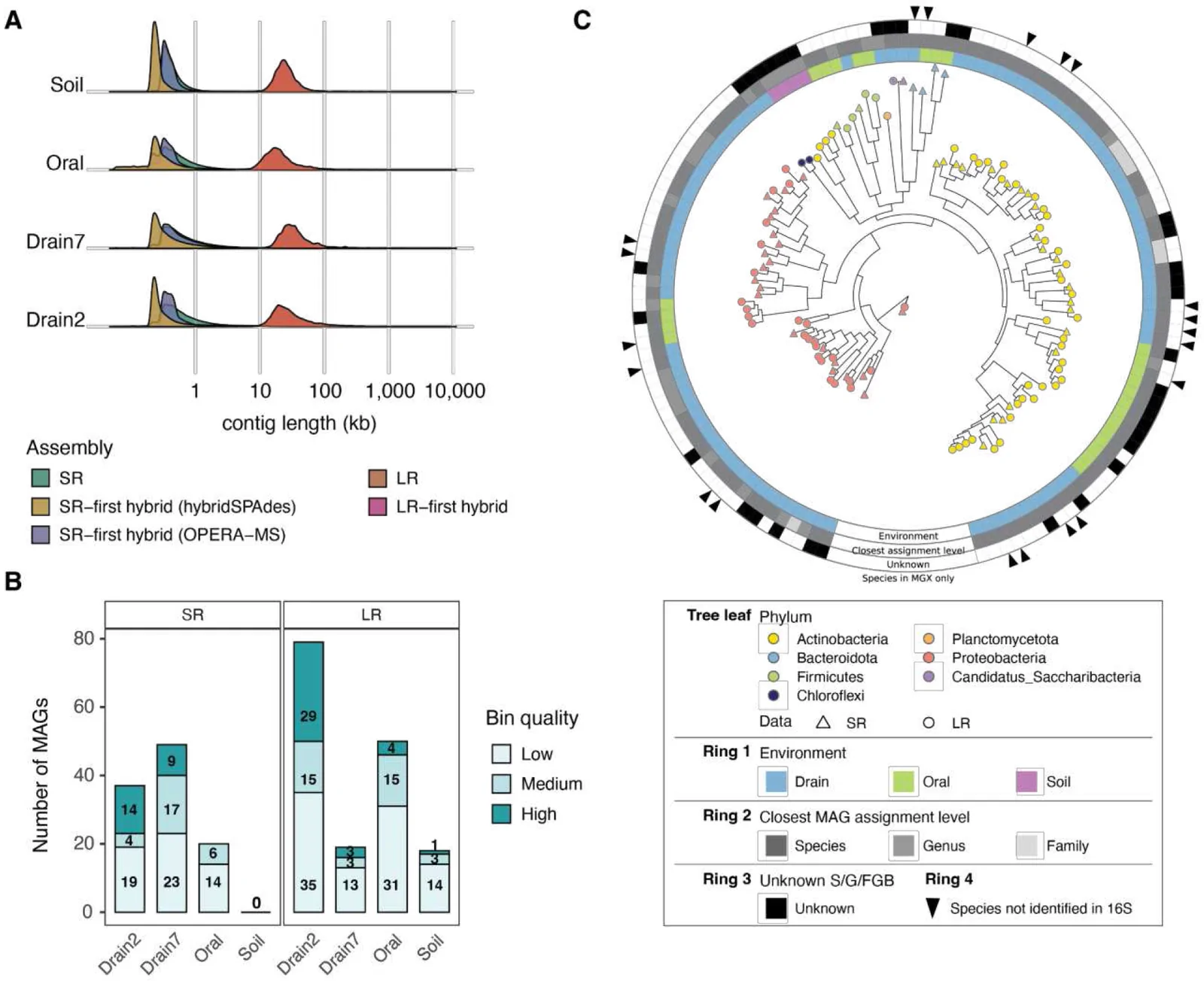
Assembly performance and MAG characteristics from SR and LR assemblies of four metagenomes. **A)** Contig length distribution (kbp) across different assembly approaches: SR assembly (MEGAHIT), LR assembly (hifiasm-meta), SR-first hybrid assembly (hybridSPAdes and OPERA-MS), and LR-first hybrid assembly/polishing (hifiasm-meta + Polypolish). These were run on two individual drain samples, one pooled oral sample (n = 8 individuals), and one pooled soil sample (n = 4 locations). **B)** Number of low-, medium-, and high-quality MAGs recovered from SR and LR assemblies, as defined by CheckM2 [37] completeness and contamination metrics (high-quality: >90% completeness, <5% contamination; medium-quality: >50% completeness, <10% contamination). **C)** Phylogenetic tree of medium and high-quality MAGs recovered from SR and LR assemblies. The phylogeny was reconstructed using PhyloPhlAn 3 from 123 medium and high-quality MAGs obtained from both SR and LR assemblies. MAGs are assigned to a known (kSGB, kGGB, or kFGB) genome bin when possible vs. unknown (uSGB, uGGB, or uFGB), i.e. knowns containing at least one taxonomically assignable genome. Triangles on the outermost ring highlight novel species recovered from metagenomic assemblies that were not identified in the FL-16S data from this study.

**Figure 5:**
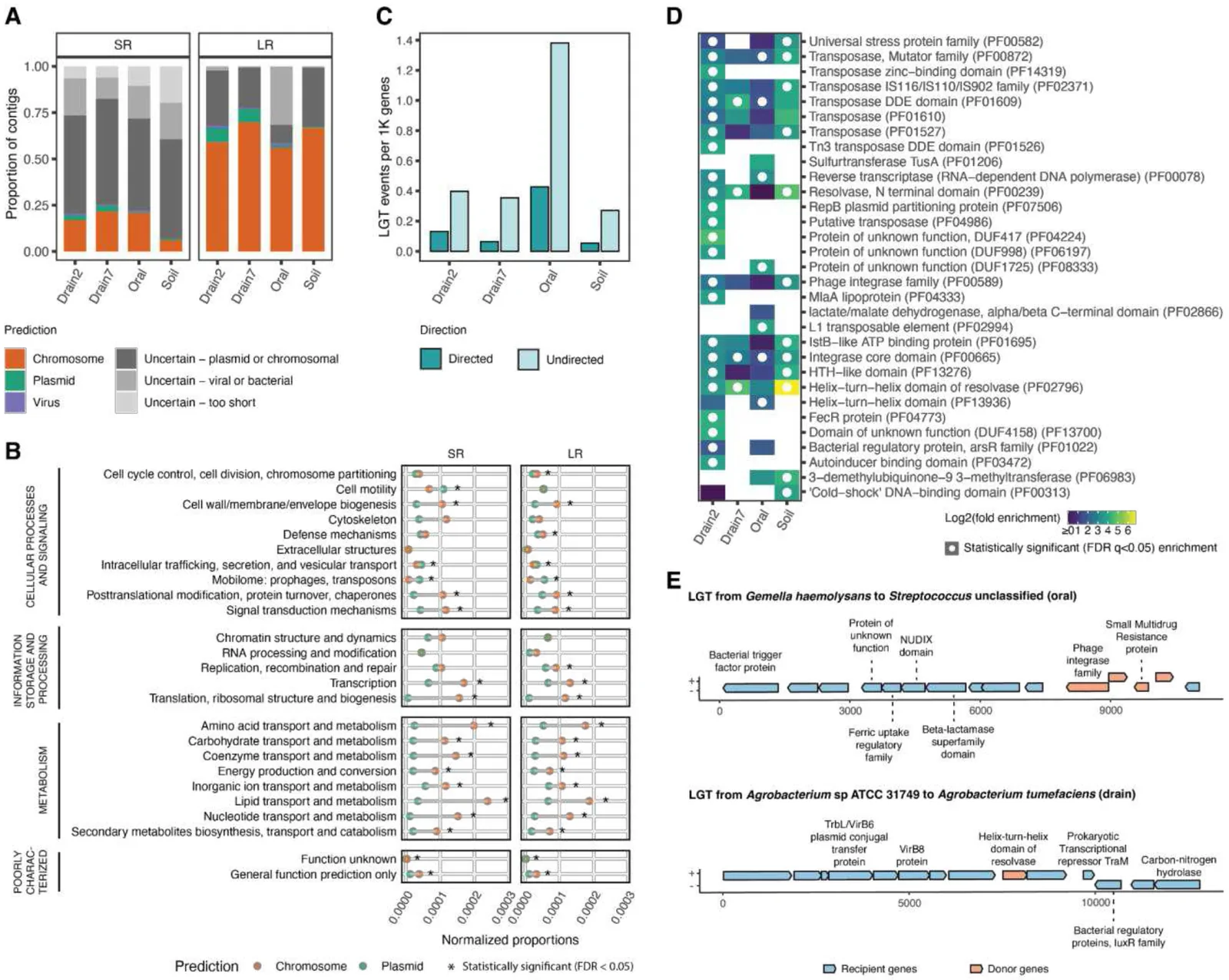
Mobile genetic elements in childcare microbiome assemblies. **A)** Classification of contigs assembled from SR and LR metagenomes as bacterial chromosomal, plasmid, viral, or uncertain based on gene content using viralVerify [39]. **B)** Molecular functions associated with plasmid contigs. Genes from chromosomal and plasmid contigs were annotated by Prokka [45] using COG functional categories [40]. The proportion of contigs annotated in each dataset was normalized by COG category size to account for inherent heterogeneity of the COG database. Functional enrichment significance was determined using Fisher's exact test with Benjamini-Hochberg FDR correction for multiple testing. **C)** Rates of undirected and directed lateral gene transfer (LGT) events across four metagenomes profiled by WAAFLE [41], normalized to total assembly size per 1,000 genes. For each sample, both directed events (where donor and recipient clades were clearly identifiable from gene adjacency) and undirected events (where donor and recipient clades were unknown) are shown. **D)** Molecular functions associated with LGT events. Fold enrichments for Pfam [46] domains in LGT-containing contigs relative to non-LGT contigs (those without detected LGT events). Significance testing was performed as in panel B). The top 10 significantly enriched domains (FDR < 0.05) by mean log2 fold enrichment are displayed. **E)** Representative LGT events from oral and drain metagenomes. Top: An inter-order phage-mediated transfer of a multidrug resistance gene from *Gemella haemolysans* to an unknown *Streptococcus* species. Bottom: An intra-genus acquisition of a DNA-binding domain of resolvase by *Agrobacterium tumefaciens* positioned between Type IV secretion system components, suggesting conjugation-facilitated gene transfer. WAAFLE has high precision for intra-genus and more distant transfer events (<0.5% overall false positives), supporting these detections [41].

**Figure 6:**
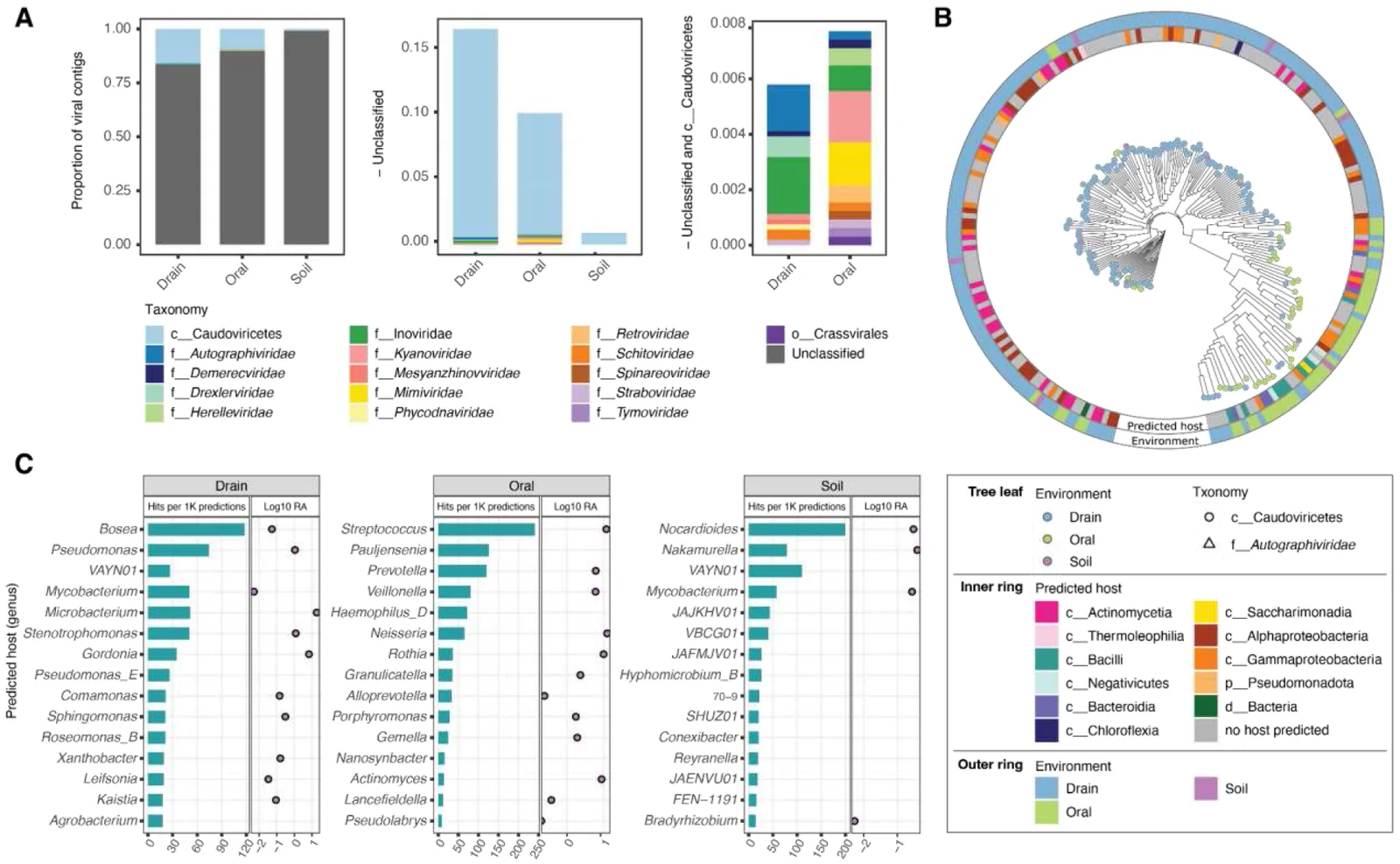
Viruses and phage-host relationships from assembled contigs. **A)** Viral contig classification across taxonomic levels. The left panel shows the complete classification distribution; the middle panel displays the taxonomic breakdown excluding the unclassified fraction (which represents the majority); the right panel shows the finer taxonomic resolution after excluding both unclassified contigs and those belonging to the predominant *Caudoviricetes* class. **B)** Phylogenetic reconstruction of *Caudoviricetes* phages based on viral contigs longer than 10 kb. Tree leaves and the outermost ring are colored according to sampling environment. The inner ring indicates predicted host taxonomy at class level (or higher taxonomic rank when class could not be resolved). **C)** Relationship between predicted phage-host associations and metagenomic host abundance across three environments. Each facet represents a distinct environment with dual visualization: bar plots showing the normalized frequency of bacterial genera predicted as phage hosts per 1,000 predictions, alongside dot plots indicating the corresponding relative abundance of each host genus from SR taxonomic profiling. For the two drain samples, relative abundances of genera were averaged. Absent dots represent genera predicted in phage-host predictions but not profiled in the metagenomic data.

**Table 1:** Summary of paired SR and LR metagenomic sequence data.

	Short reads					Long reads				
	Illumina reads (M)	Total data (Gb)	Mean length (bp)	Max. length (bp)	Min. length (bp)	HiFi reads (M)	Total data (Gb)	Mean length (bp)	Max. length (bp)	Min. length (bp)
Drain2	76.37	10.24	134	151	46	2.07	14.85	7,183	24,774	93
Drain7	116.31	14.45	124	151	46	0.1	0.93	9,313	27,449	84
Oral	13.47	1.87	121	151	20	0.45	2.27	5,076	29,157	83
Soil	31.56	3.8	139	151	74	0.37	2.63	7,083	30,566	78

**Table 2:** Assembly statistics of contigs produced by SR, LR, and hybrid assemblers.

	Short-read assembly				Long-read assembly				Hybrid assembly			
	Drain 2	Drain 7	Oral	Soil	Drain 2	Drain 7	Oral	Soil	Drain 2	Drain 7	Oral	Soil
# contigs	203,003	209,960	213,559	167,573	5,050	1,324	4,758	2,174	159,916	247,180	196,500	132,719
# contigs ≥ 2,500 bp	14,754	16,629	6,022	110	5,049	1,324	4,758	2,174	8,153	14,130	5,934	1,677
# contigs ≥ 10 kbp	3,363	3,763	256	0	5,007	1,323	4,418	2,164	3,519	3,381	2,027	410
# contigs ≥ 50 kbp	353	467	0	0	963	269	467	110	715	394	213	20
Largest contig (Mbp)	0.34	0.60	0.05	0.01	9.50	5.09	2.35	2.74	3.81	1.64	0.28	0.12
N50	3,003	3,513	812	445	296,938	58,084	43,906	29,622	53,210	2,758	2,022	446
L50	12,057	11,200	43,405	60,084	101	209	564	610	671	12,483	6,781	34,138
Total length (Mbp)	267.60	299.97	149.07	77.20	374.36	69.20	159.00	67.25	314.05	299.00	155.16	69.96
Total length of contigs ≥ 10 kbp (Mbp)	89.00	108.85	3.72	0	374.01	69.19	156.23	67.17	219.25	106.09	55.47	8.58
Total length of contigs ≥ 50 kbp (Mbp)	29.86	39.60	0	0	272.78	37.85	74.98	15.66	159.34	44.92	18.25	1.42
% short reads mapped to contigs (≥ 2,500 bp)	90.16%	91.98%	49.35%	0.17%	96.28%	78.75%	83.71%	11.21%	95.30%	87.49%	82.22%	5.39%
