# Supplementary material for: Microbial transmission and ecology of human and environmental microbial communities in childcare centers
Source: Res Sq. 2026 Sep 10:rs.3.rs-10207669. Preprint. [Version 1] doi: 10.21203/rs.3.rs-10207669/v1 (PMC13622856; doi:10.21203/rs.3.rs-10207669/v1)
Supplement: Supplementary file 1 [file NIHPPrs10207669v1-supplement-1.pdf]

Supplementary materials

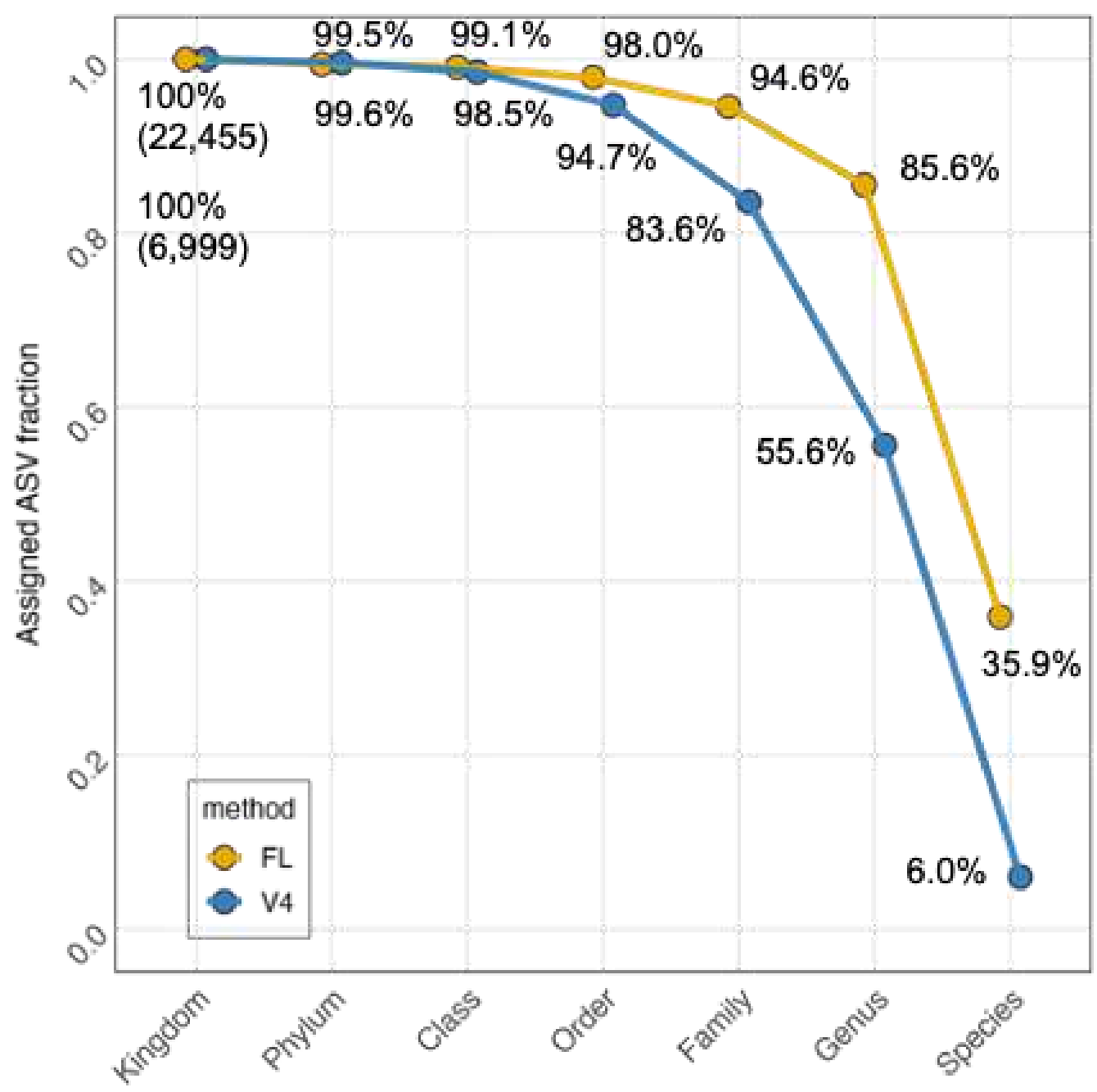

Figure S1: Comparison of taxonomic resolution between full-length 16S and V4 amplicons. Fraction of ASVs successfully assigned at each taxonomic rank (Kingdom through Species), comparing full-length 16S (FL) and in silico V4 amplicons. Both methods achieve near-complete assignment at higher ranks (Kingdom through Class) but diverge progressively at Family and below, with FL-16S resolving 35.9% of ASVs to the species level compared to 6.0% by V4 amplicons.

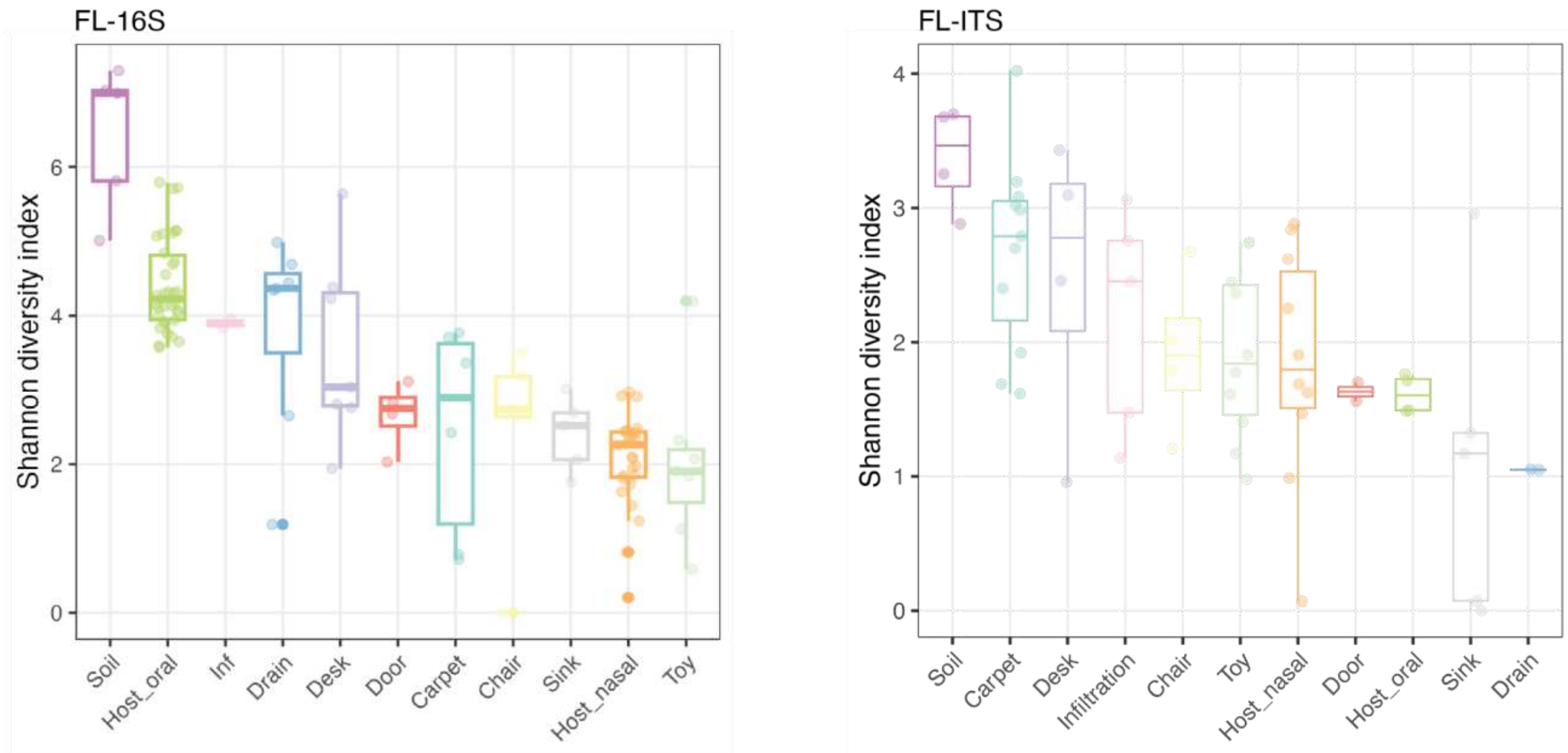

Figure S2: Alpha diversity across sample types in FL-16S (left) and FL-ITS (right) datasets. Shannon diversity index distributions across all sample types. A) Bacterial alpha diversity (FL-16S) was highest in soil samples (median Shannon index = 6.99), followed by oral, infiltration, and drain samples, with toy samples showing the lowest diversity (median Shannon index = 1.90). B) Fungal alpha diversity (FL-ITS) was similarly highest in soil, followed by carpets, with the lowest diversity in sink and drain samples. Only 61 of 177 samples yielded sufficient fungal DNA for amplification and sequencing.

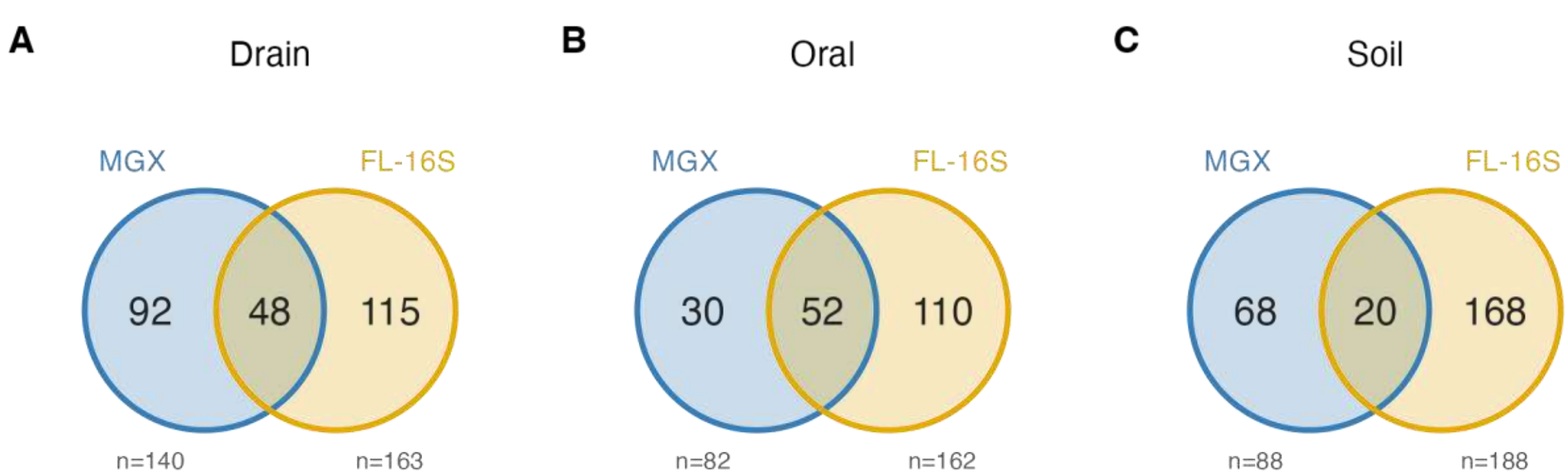

Figure S3: Species-level overlap between FL-16S amplicons and shotgun metagenomics (MGX) across matched sample types. Venn diagrams show the number of species detected exclusively by FL-16S, exclusively by MGX, or by both methods, for A) drain, B) oral, and C) soil samples. Total species detected per method (n) are shown below each circle.

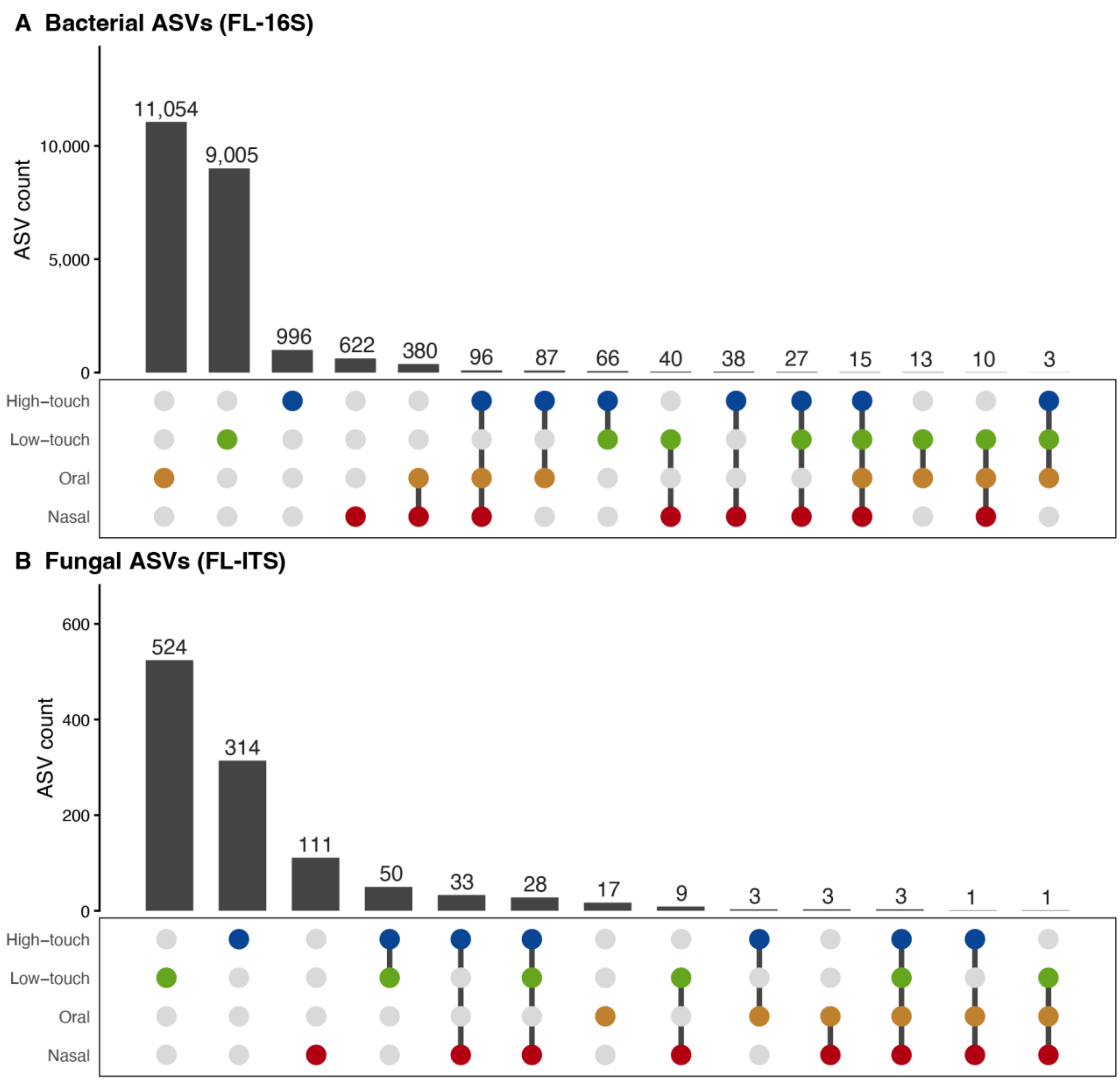

Figure S4: UpSet plot of bacterial and fungal ASV sharing across sample types. A) Bacterial ASVs (FL-16S). B) Fungal ASVs (FL-ITS). Each bar represents the number of ASVs exclusive to or shared among the indicated combination of sample types (High-touch surfaces, Low-touch surfaces, Oral, Nasal). Colored dots indicate set membership; gray dots indicate absence. Intersections are ordered by decreasing size.

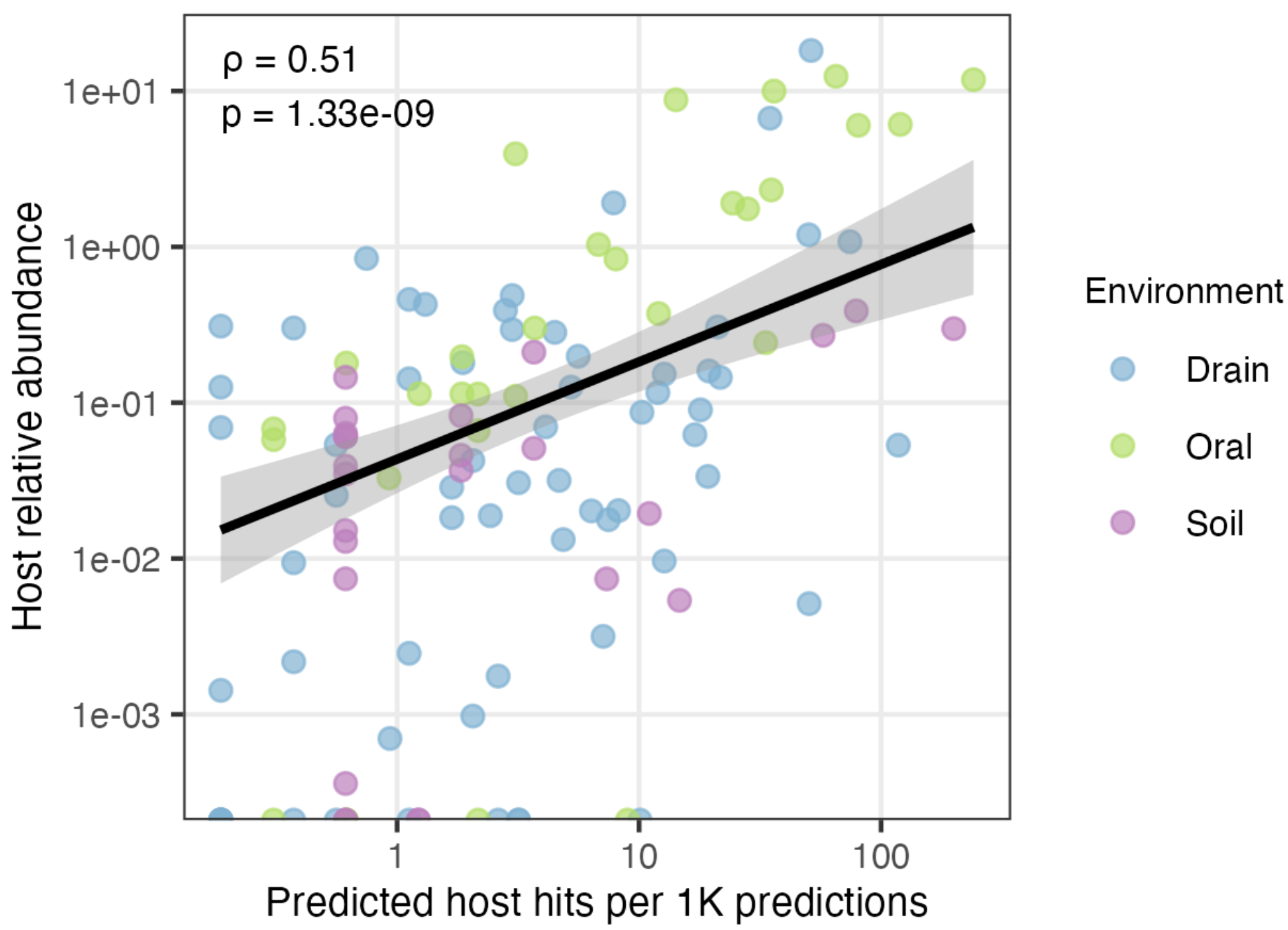

Figure S5: Correlation between the frequency of host genus predictions and host relative abundance. Scatter plot showing the relationship between the predicted host genus frequency (normalized frequency of bacterial genera predicted as phage hosts per 1,000 predictions) and the relative abundance of that genus across drain, oral, and soil metagenomes. A strong positive correlation was observed (Spearman $\rho = 0.60$, $p < 0.001$), suggesting that host availability is a principal driver of phage–bacteria population dynamics.

Additional file 1 (.xlsx): Sample overview.

Additional file 2 (.xlsx): Sample metadata.

Additional file 3 (.zip): Amplicon feature tables (four tab-separated files). 16S_ASV_table.tsv, ITS_ASV_table.tsv, 16S_OTU_table.tsv, ITS_OTU_table.tsv.

Additional file 4 (.xlsx): Number of shared ASVs between host and environmental samples.

Additional file 5 (.xlsx): ASV contribution of bacterial (n = 28) and fungal (n = 23) species shown in Figure 3A (union of most prevalent species per touch-frequency group and most abundant species per sample type).

Additional file 6 (.xlsx): Metagenomic assembly results. Summary statistics of metagenomic assembly (Sheet 1), medium- and high-quality MAG assignment (Sheet 2).

Additional file 7 (.tsv): Contig classification output.

Additional file 8 (.tsv): WAAFLE LGT profile.

Additional file 9 (.tsv): Molecular functions (Pfam domains) enriched in LGT-containing contigs relative to background contigs.

Additional file 10 (.xlsx): Viral analysis. Viral taxonomic profile (Sheet 1), phage host prediction (Sheet 2).

Additional file 11 (.tsv): Metagenomic taxonomic profile.
